# Limited increases in Arctic offshore oil and gas production with climate change and the implications for energy markets

**DOI:** 10.1038/s41598-024-54007-x

**Published:** 2024-03-20

**Authors:** Ying Zhang, Siwa Msangi, James Edmonds, Stephanie Waldhoff

**Affiliations:** 1 Joint Global Change Research Institute, Pacific Northwest National Laboratory, 5825 University Research Ct, College Park, MD 20740 USA; 2 Economic Research Service, US Department of Agriculture, 1400 Independence Ave, SW, Washington, DC 20250 USA

**Keywords:** Arctic offshore oil and gas, Sea ice, Climate change, Energy market, Human-Earth system, Integrated assessment model, Climate and Earth system modelling, Climate-change impacts, Projection and prediction, Climate change, Climate-change mitigation

## Abstract

Climate change impacts on sea ice thickness is opening access to offshore Arctic resources. The degree to which these resources are exploited will depend on sea-ice conditions, technology costs, international energy markets, and the regulatory environment. We use an integrated human-Earth system model, GCAM, to explore the effects of spatial–temporal patterns of sea-ice loss under climate change on future Arctic offshore oil and gas extraction, considering interactions with global energy markets and emission reduction scenarios. We find that under SSP5, a “fossil-fueled development” scenario, the effects of sea-ice loss are larger for Arctic offshore oil production than gas. Under SSP5, future extraction of Arctic offshore oil and gas through 2100 adds roughly 0.8–2.6 EJ/year to oil and gas markets but does not have large impacts on global oil and gas markets. Surprisingly, a low-carbon scenario results in greater Arctic offshore oil production to offset the more emissions-intensive unconventional oil production.

## Introduction

Climate change has led to Arctic sea-ice thinning. The loss of sea ice will open new regions in the Arctic for human activities such as oil and gas extraction. The United States Geological Survey (USGS) has assessed the undiscovered oil in the Arctic to be 500 Exajoules (EJ)^1^, which is approximately three times the world’s crude oil production in 2015^2^. The undiscovered gas in the Arctic, including natural gas liquids, is assessed to be 1850 EJ^1^—about 11 times the world’s gross gas production in 2015^3^. Approximately 84% of the assessed Arctic oil and gas potential is offshore^1^, where 22% and 69% of the offshore oil and gas, respectively, is located in the Russian Arctic; 40% and 12% in the US Arctic; 21% and 10% in the Greenland Arctic; 13% and 5% in the Canadian Arctic; and 4% and 4% in the Norwegian Arctic.

Climate models from the sixth phase of the Coupled Model Intercomparison Project (CMIP6)^4^ agree on an overall trend of sea ice thinning in the Arctic over the next few decades, particularly under high warming scenarios^5^. This will increase access to the prospective offshore oil and gas resources that have been cost prohibitive to extract under historical sea-ice conditions. Thinner sea ice or completely ice-free conditions would enable the use of lower cost offshore extraction technologies^6^, leading to a potential increase in Arctic oil and gas production under the changing climate.

Spatial and temporal projections of sea-ice thickness and extent are highly dependent on future warming conditions^5^. Even within a single warming scenario, projections vary across climate models and ensemble runs. Moreover, the spatial–temporal variability of sea-ice conditions across the Arctic will affect where and when it will be technically and economically viable to extract the underlying oil and gas resources.

While there are other crucial determinants of resource extraction potential in the Arctic, such as national energy security objectives and tax incentives, we focus on the climate, technical, and economic aspects in projecting the future of Arctic offshore oil and gas production and assume the other determinants to be *business as usual* (i.e., remain at current levels and trends). Using an integrated human-Earth system model, we capture the regional market interactions between Arctic and non-Arctic oil and gas resources, as well as those with other energy sources under climate and socioeconomic changes.

To our knowledge, no previous study has performed an integrated modeling of changes in future Arctic offshore oil and gas production due to climate impacts and energy market interactions with the rest of the globe. Specifically, we explore the impacts of global climate change on Arctic sea-ice conditions and its implications for Arctic offshore oil and gas production as well as global energy markets at large, using a dynamic, global, integrated, human-Earth system model. The model is a modified version of the Global Change Analysis Model (GCAM)^7^, in which we explicitly break out Arctic offshore oil and gas resources for the five GCAM Arctic regions, based on the 60 geological Assessment Units. We explore the long-term future of Arctic offshore oil and gas production through the end of the century considering the individual and interactive effects of climate change on sea-ice loss and scenarios that achieve a low-carbon future.

## Methods

### Sea-ice thickness, resource potential, and extraction costs

To model climate change impacts on Arctic offshore oil and gas production, we first select global climate models in CMIP6^4^ that are shown to perform the best in historical simulations of Arctic sea ice compared to observations^5^. We then obtain the gridded data of monthly sea-ice thickness projections under different climate warming scenarios from those model ensembles^8^. In total, we obtain 13 model ensembles (Table S1) under two Representative Concentration Pathways (RCP) scenarios (i.e., RCP2.6 and 8.5). RCPs are trajectory scenarios, which include emissions and concentrations of greenhouse gases (GHGs), aerosols and chemically active gases, as well as land use/land cover changes over time^9,10^. The numbers, 2.6 and 8.5, represent the approximate radiative forcing, in W/m^2^, in 2100 under the respective RCP, resulting in different levels of climate change.

The USGS estimated the undiscovered oil and gas potential in the Arctic at the Assessment Unit (AU) level, which is a sub-unit of hydrocarbon basins with similar geological properties^1,11^. A total of 53 *offshore* AUs are selected, and the associated AU-level oil and gas resource potentials are implemented in our model. A map the offshore AUs are provided in Fig. S1. The associated oil and gas potential at each AU is provided in Table S2.

To estimate the extraction costs for exploiting the oil and gas potential in each AU in response to the sea-ice conditions, we first average the gridded sea-ice thickness at the AU level. We then develop cost functions which depend on the temporal window within which the AU-level monthly sea-ice thickness falls under a critical threshold necessary for viable offshore extraction technologies to be installed and used; that is, an AU is considered not tappable when sea-ice thickness is greater than 1.5 m; an AU is tappable by *subsea* offshore extraction technology when there are three consecutive months with sea-ice thickness less than 1.5 m; and when the monthly sea-ice thickness is below 1.5 m and will no longer exceed 1.5 m, a cheaper technology, *floating*, becomes viable^6^ (Fig. S2). More information on estimating the extraction costs can be found in Appendix S1 (Figs. S3–S5).

### Arctic offshore oil and gas in GCAM

We use the integrated human-Earth system model, GCAM^7^, to couple Arctic offshore oil and gas production to global oil and gas markets, which in turn are coupled to the larger global socioeconomic, energy, agriculture, land, water, and climate systems. The model simultaneously solves all energy, agriculture-land-use, and water markets in a dynamic-recursive approach. For more details, please refer to the model documentation available at https://jgcri.github.io/gcam-doc/.

In this study, the AU-level Arctic offshore oil and gas potential along with the extraction costs derived given the sea-ice thickness in each AU are incorporated into GCAM. As 32 regions are modeled globally in GCAM’s energy and socioeconomic systems, the 53 offshore AUs overlap with five of the 32 regions (i.e., USA, Canada, Russia, Norway as part of European Free Trade Association (EFTA), and Greenland as part of EU-15; Table S3), resulting in 60 AU-region combinations (Table S2).

The newly developed representation of AU-specific Arctic offshore oil is nested within the GCAM oil resource-reserve production structure, where non-Arctic crude oil and unconventional oil are also nested. Similarly, AU-specific Arctic offshore gas and non-Arctic gas are nested under GCAM’s natural gas commodity category. The total oil and gas are then processed in the downstream energy transformation and end-use sectors to meet regionally differentiated market demand. They are also traded globally as primary energy sources with considerations of preferences between domestic and imported commodities^12^. The extraction of nested resources depends on the resource supply curves and the endogenously solved regional market prices. Once the economically attractive resources are extracted and added to reserves, production out of each reserve occurs over time.

GCAM is calibrated over historical periods spanning from 1975 to the model base year of 2015, from which it runs at a 5-year time step (i.e., 2020, 2025, …) through 2100. The GCAM resource supply curves for Arctic offshore oil and gas are defined by five-year averages of the annual extraction costs centered around each GCAM period and the associated USGS estimated quantities of resource potential across AUs (Figs. S6–S10). As the sea-ice thickness changes over time and space, the corresponding resource supply curve shifts over the projection periods.

Additionally, the extraction costs for climate model ensembles under RCP scenarios in the base year (i.e., 2015) are averaged to obtain a unified base-year resource supply curve. We then incorporate the climate change impacts on sea-ice thickness, projected by these climate model ensembles, using relative ratios of the associated extraction costs in the future periods in comparison to the same base-year cost. In this way, we start with the same base year cost and retain the variation of extraction costs based on different projections of the climate model ensembles for the future periods. We include more details in the Appendix S2.

### GCAM model scenarios

We analyze five scenarios, defined by three future sets of sea-ice conditions (Table 1) and two GHG emission pathways, to study the individual and interactive effects of climate change and decarbonization efforts on the spatial and temporal patterns of future Arctic offshore oil and gas production.

**Table 1:** GCAM model scenarios. Note that all scenarios are run under GCAM’s SSP5, “fossil-fueled development”.

	Climate change	Low-carbon future
Ref	No change of sea-ice conditions from present	No low-carbon scenario
RCP2.6	Change of sea-ice conditions under RCP2.6	
RCP8.5	Change of sea-ice conditions under RCP8.5	
LowC	No change of sea-ice conditions from present	RCP2.6 in 2100
LowC|2.6	Change of sea-ice conditions under RCP2.6	

Scenario RCP2.6, RCP8.5, and LowC|2.6 include 13 climate ensembles for each.

The first three scenarios explore the role of sea-ice conditions on Arctic offshore oil and gas extraction: a Reference (“Ref”), with no change in sea ice from present, and changes in sea-ice thickness under RCPs 2.6 and 8.5 (“RCP2.6” and “RCP8.5”), which define the lower and upper boundary of climate change impacts on sea-ice thickness explored in CMIP6. The fourth scenario is designed to explore the discrete impact of changes in the energy system in a low-carbon future scenario (“LowC”), which achieves a radiative forcing of 2.6 W/m^2^ in 2100 (RCP2.6), while sea-ice conditions remain the same as the reference. The final scenario explores the joint effects of climate change impacts on sea-ice thickness under RCP2.6 and the emissions reductions required to achieve this pathway (“LowC|2.6”). Each of the scenarios with climate change impacts has 13 ensembles corresponding to the climate model ensembles. All scenarios (including ensembles) are run with the newly developed representation of AU-specific Arctic offshore oil and gas resource-reserve technologies in GCAM.

Note that Shared-Socioeconomic Pathway 5 (SSP5) in GCAM is used as the baseline for all model scenarios (https://jgcri.github.io/gcam-doc/ssp.html). We use SSP5 because, in this scenario, fossil fuel development is intensive and therefore could lead to the most noticeable changes, if any, in the future Arctic offshore oil and gas production as sea ice thins. The SSPs do not include major energy system transitions. However, they can be paired with the transition pathways that achieve lower radiative forcing targets, such as the RCP2.6 used in this study. This enables exploration of the implications of emissions reductions scenarios under the SSP5. Under this Ref scenario, the model is instructed to find a pathway that reaches the target in 2100 through carbon taxes (placing a price on emissions). The price is transmitted through the modeled systems, which affects the production and consumption across markets. For example, carbon-intensive resource production would be reduced due to the carbon tax because it would cost more to produce such resources than a scenario without the carbon tax.

The LowC scenario employed in this study uses a pathway target of RCP2.6 to be reached in 2100. In this scenario, GHG reductions through 2035 are adjusted to represent current regulations, after which the model finds a pathway to reach the target in 2100. The resultant carbon tax pathway under LowC and LowC|2.6 are shown in the Fig. S11. More information on the pathway target in GCAM can be found at https://jgcri.github.io/gcam-doc/policies.html.

## Results

### Sea-ice thickness

**Figure 1:**
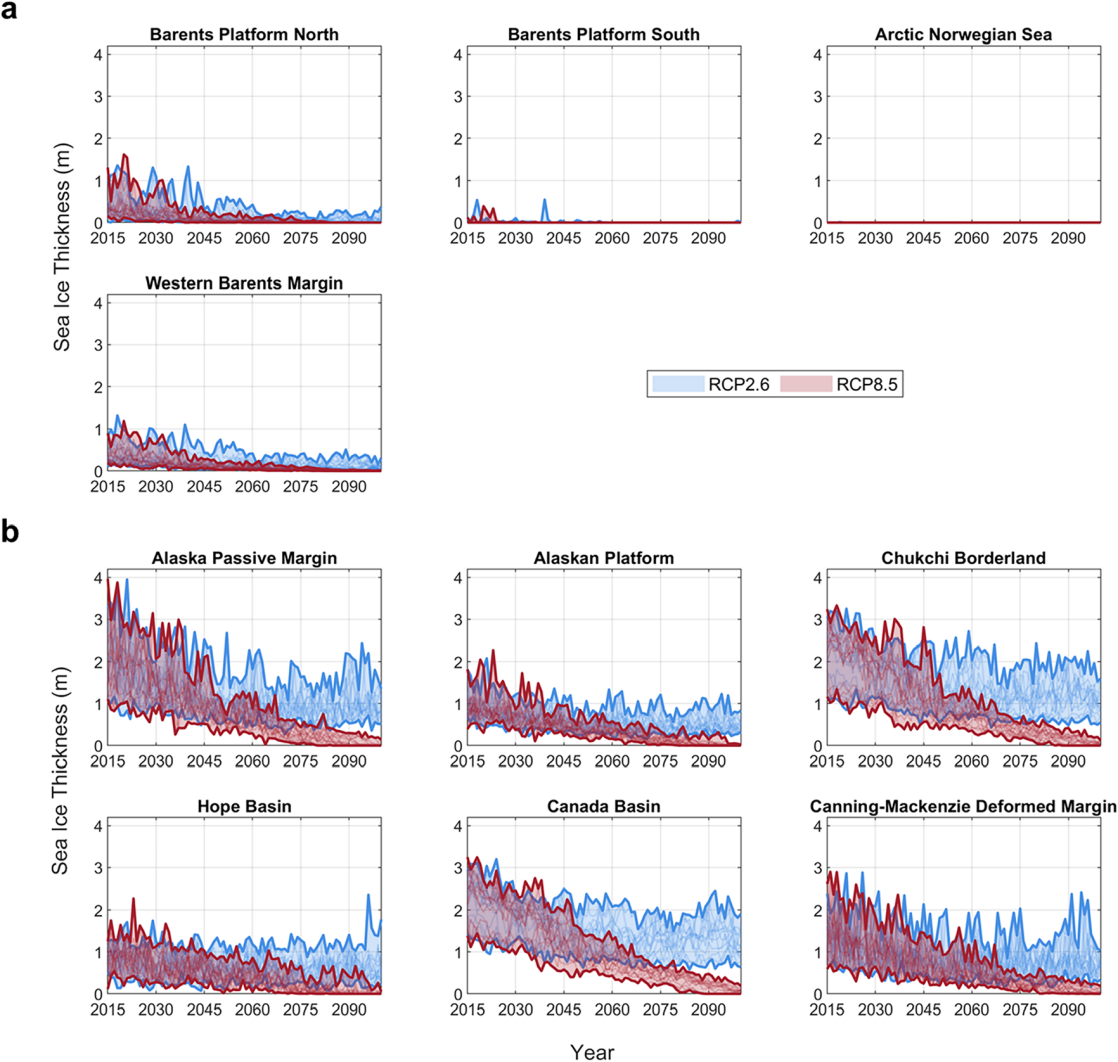
Annual sea-ice thickness over 2015–2100 across Assessment Units (AUs) in (**a**) Norway and (**b**) USA (See other regions in Figs. S12–S14), based on projections from CMIP6 model ensembles under RCP2.6 and 8.5. Each RCP category is shown in a single-color band bounded by the maximum and minimum value of all ensembles over years.

Both RCP2.6 and 8.5 project a trend of decreasing annual sea-ice thickness through 2100 at the AU level (Fig. 1 and Figs. S12–S14). The rate of decrease is larger under the warmer climate scenario, RCP8.5, leading to ice-free conditions (sea-ice thickness equal to zero) in 2100 in almost all AUs, with less uncertainty across the model ensembles than the lower radiative forcing scenario, RCP2.6.

However, there is large spatial variability in sea-ice thickness across the AUs earlier in the time horizon and under RCP2.6. The annual sea-ice thickness is generally lower in AUs in Norway than other regions, particularly in Norway’s Barents Platform South and Arctic Norwegian Sea, where the AU-level sea-ice thickness is projected to be zero over the entire 2015–2100 period in almost all model ensembles (Fig. 1a). Sea-ice thickness spans wider ranges across model ensembles and AUs in the USA, where the Alaskan platform and Hope basin have generally lower sea-ice thickness than in other AUs (Fig. 1b).

### Arctic offshore oil and gas extraction costs

**Figure 2:**
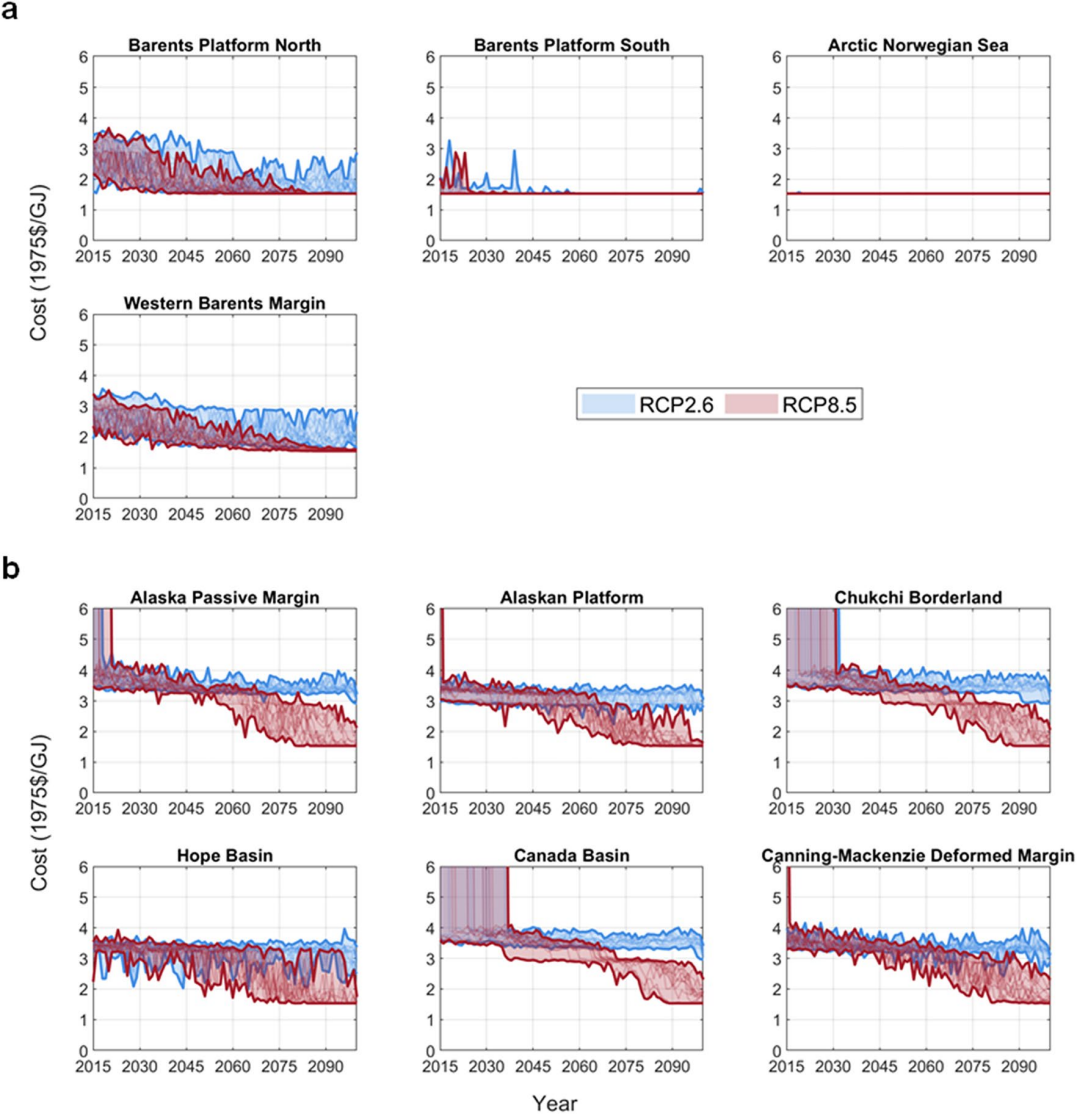
Extraction costs for Arctic offshore oil over 2015–2100 across Assessment Units (AUs) in (**a**) Norway and (**b**) USA (see other regions and the costs for Arctic offshore gas in Figs. S15–S22), given sea-ice thickness projections from CMIP6 model ensembles under RCP2.6 and 8.5. Each RCP category is shown in a single-color band bounded by the maximum and minimum value of all ensembles over years. Costs beyond the y-axis upper limit indicate that the assessment unit is untappable at that time. The gigajoule (GJ) is equal to 10^9^ J and approximately 0.2 barrel of oil equivalent (BBOE).

The overall pattern of extraction costs follows the sea-ice thickness change over time; that is, the resultant extraction costs are generally lower by the end of the century than early decades and lower under RCP8.5 than RCP2.6 (Fig. 2 and Figs. S15–S22). As a result, the extraction costs are at the lower bound when sea-ice thickness is zero, making the oil and gas under the sea more economically attractive (e.g., Arctic Norwegian Sea; Fig. 2a). The extraction costs for tappable AUs are contained within the upper- and lower-bound costs for Arctic oil and gas production defined by the International Energy Agency (see Appendix S1).

### Future Arctic offshore oil and gas production

#### Regional results

**Figure 3:**
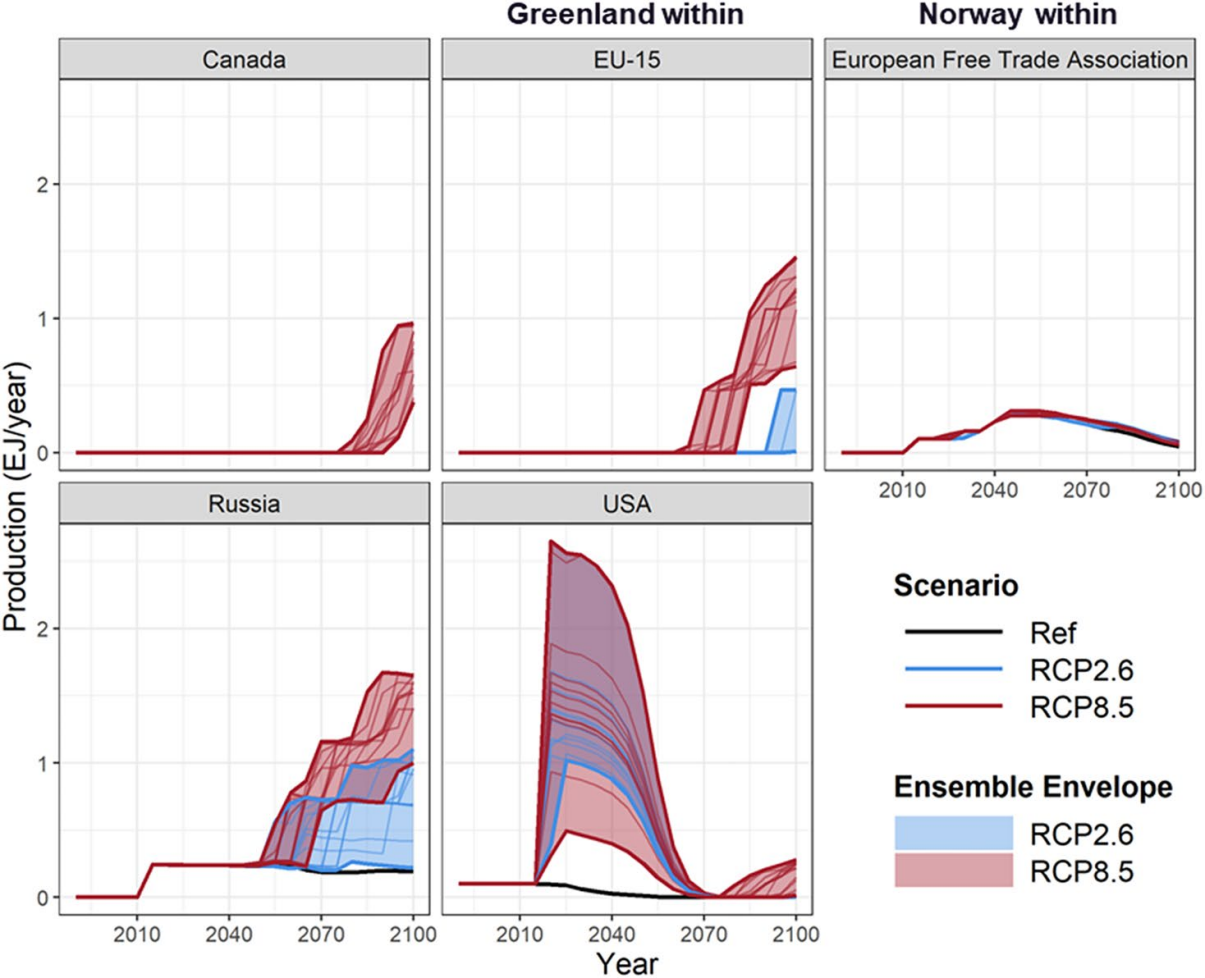
Arctic offshore oil production across regions under Ref, RCP2.6, and 8.5. The RCP2.6 and 8.5 include 13 ensembles each, and thus an ensemble envelope is shown with the maximum and minimum value being the upper and lower bound. The exajoule (EJ) is equal to 10^18^ J and approximately 0.2 billion barrels of oil equivalent (BBOE).

The potential future Arctic offshore oil production varies across regions and scenarios (Fig. 3). Under Ref sea-ice conditions, production of Arctic offshore oil begins in Norway, Russia, and USA before or around the model base year (i.e., 2015), consistent with historically observed extraction in these regions. However, our results show that under Ref, Canada and Greenland are unlikely to have economically viable production for Arctic offshore oil before the end of our simulation period (2100).

As expected, the climate change impacts on sea-ice conditions will affect the timing of future production of Arctic offshore oil, as sea-ice thickness and extent decreases, improving the economic competitiveness of these resources. Under our RCP8.5 simulations, Canada and Greenland are projected to start production around 2080 and 2070, respectively. Greenland is also projected to begin production under RCP 2.6 in 2095.

The difference between RCP 2.6 and 8.5 is minimal in terms of the timing of beginning production for Russia and the USA. However, the climate scenarios have a pronounced effect on the production quantity (Fig. 3). Under current sea-ice conditions (Ref), USA and Russia are unlikely to expand Arctic offshore oil production through 2100, while climate change leads to drastic increase of production, particularly in the USA, which sees much higher production in the near term under RCP2.6 and 8.5 than Ref. In general, RCP8.5 leads to earlier and higher production of Arctic offshore oil than RCP2.6 and Ref.

In Norway projections of production under both RCPs differ little from the Ref scenario without sea ice impacts (Fig. 3). This is due to relatively small impact of climate change on sea-ice conditions in Norway (Fig. 1a) and thus the extraction costs (Fig. 2a), particularly at the AUs with relatively large oil resource potential, such as Barents Platform South and Arctic Norwegian Sea (Table S2).

**Figure 4:**
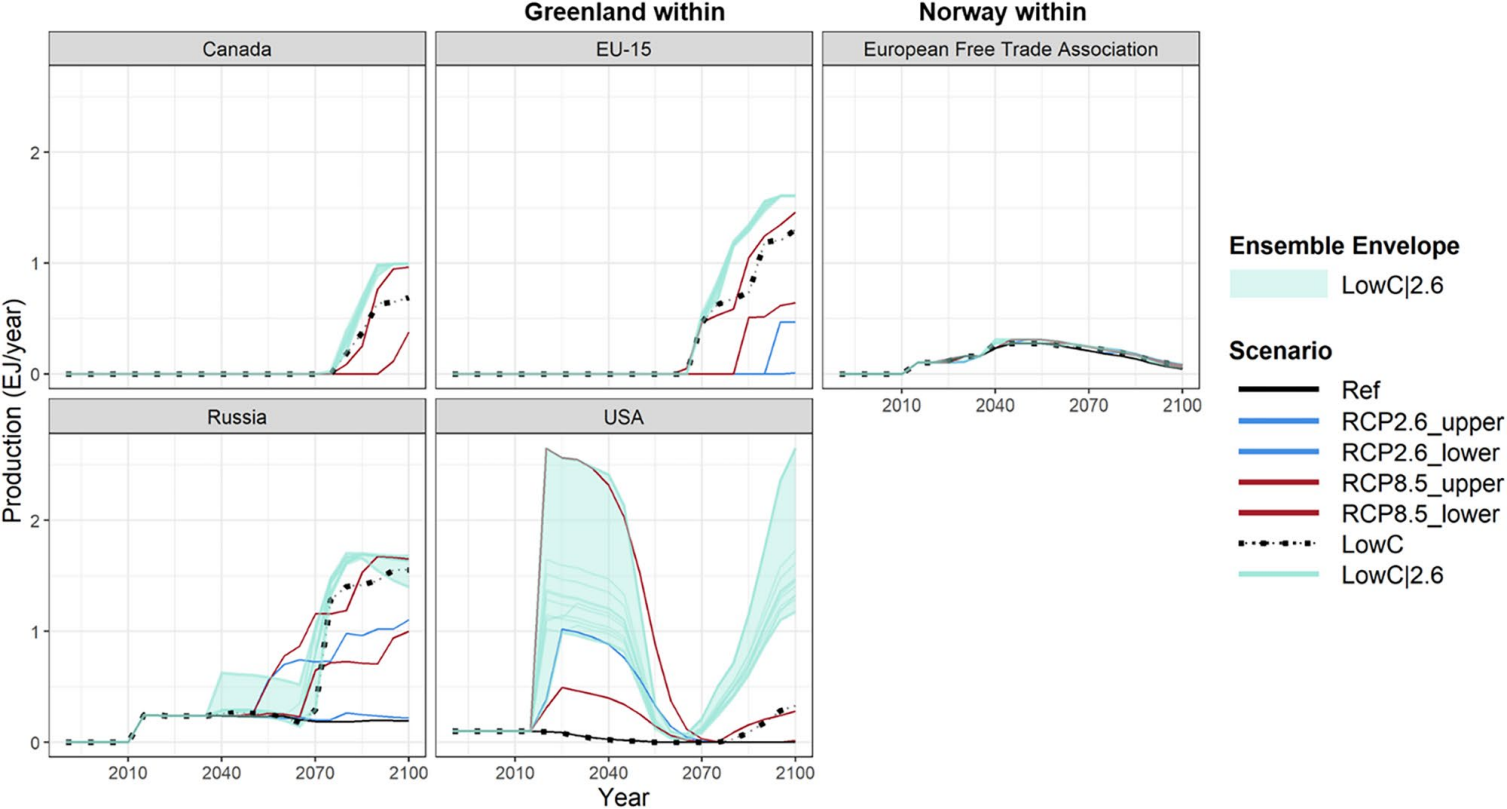
Arctic offshore oil production across regions under LowC and LowC|2.6, in comparison to Ref, and the lower and upper bounds of RCP2.6 and 8.5. The LowC|2.6 includes 13 ensembles, with the ensemble envelope defined by the maximum and minimum value being the upper and lower bound. The exajoule (EJ) is equal to 10^18^ J and approximately 0.2 billion barrels of oil equivalent (BBOE).

The regional Arctic offshore oil production under LowC scenario is generally higher than Ref, particularly towards the end of the century (Fig. 4). This is because Arctic offshore oil is relatively clean and can replace the unconventional oil production to meet the overall oil demand, as non-Arctic crude (conventional) oil is gradually depleted during the last few decades approaching 2100 (see more in the section “Effects on global oil and gas markets”). Sea-ice thinning leads to even higher Arctic offshore oil production under LowC|2.6, which is comparable with the production under RCP8.5, due to the combined effects of lower extraction costs with thinning sea ice and the need for comparatively clean oil under the RCP2.6 scenario.

Currently, only Norway produces Arctic offshore gas. Under the scenarios explored here, Arctic offshore gas production continues to remain exclusively in Norway (Fig. S23). The differences in Arctic offshore gas production, compared to oil, are due to relatively low costs of non-Arctic natural gas, which makes the Arctic offshore gas less economically competitive than Arctic offshore oil.

Like oil, the Arctic offshore gas production in Norway is also associated with relatively small uncertainties across scenarios. This indicates that the climate change impacts on sea-ice conditions and human efforts to reach a low-carbon future are not likely to have a large effect on Norway’s Arctic offshore oil and gas production. Overall, the projection shows that Norway’s Arctic offshore gas production increases in the near term, and then decreases to zero by 2100 (Fig. S23).

#### Assessment unit-level results

**Figure 5:**
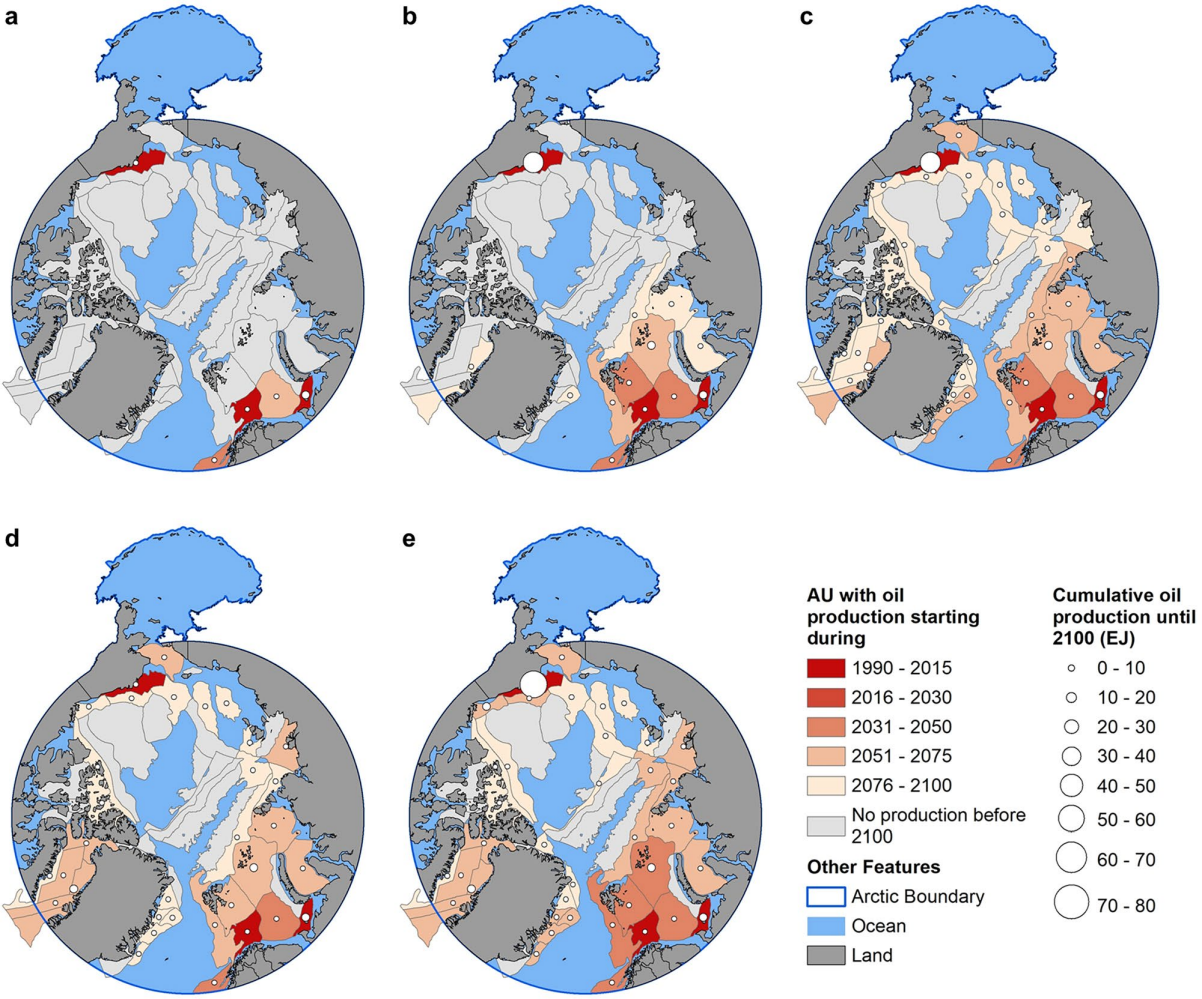
Arctic offshore oil production at the Assessment Unit (AU) level under (**a**) Ref, (**b**) RCP2.6, (**c**) RCP8.5, (**d**) LowC, and (**e**) LowC|2.6. For scenarios with multiple ensembles, the production is shown using ensemble mean. The color shows when the production first started or is projected to start. The size of the circle shows all-time cumulative offshore oil production until 2100 in that AU. The exajoule (EJ) is equal to 10^18^ J and approximately 0.2 billion barrels of oil equivalent (BBOE).

Offshore oil production levels are projected to start at various periods for different AUs, which vary under different scenarios. Under Ref, the sea-ice thickness does not change relative to the present and the viable extraction technologies in each AU do not change, so increases in production are driven only by increasing demand and the resulting increases in price. Thus, under Ref, only two additional AUs begin offshore oil production by 2100, beyond the three that have historically been producing oil. Other than one AU at the north of Alaska, all other producing AUs under Ref are located at the Norway and Northwest Russia side (Fig. 5a).

Under warming scenarios, the sea-ice thickness is higher traveling in a northeast direction at the Norway and Russia side of the Arctic, and the sea-ice thickness is generally higher at the other side of the Arctic, as shown by the 13 CMIP6 ensemble members (Fig. S24). In RCP2.6, production begins in most of the offshore AUs in Norway and Northwest Russia (Fig. 5b). Under RCP8.5, the producing AUs further extend to a large part of the USA, Canada, and Greenland side of the Arctic, although many of the AUs are projected to start the production towards the end of the century (Fig. 5c).

Under LowC, all AUs in the Baffin Bay between Canada and Greenland show positive production levels by 2100 (Fig. 5d). This production begins after 2050 when non-Arctic crude (conventional) oil is gradually depleted, and unconventional oil production is relatively more expensive under LowC due to its associated higher emissions and the cost of carbon emissions in this scenario. Arctic offshore oil, which has lower emissions than unconventional oil, becomes a more economical choice. This effect is amplified when the joint effects of climate change and low-carbon energy system transitions are modeled, as shown by more AUs producing Arctic offshore oil under LowC|2.6 (Fig. 5e) than the combined producing AUs under LowC and RCP2.6 (Fig. 5b,d). This is attributed to the simultaneous occurrence of two conditions: the decrease in sea-ice thickness, which reduces the cost of extracting Arctic offshore oil, and the reduction in emissions, which generates an increased demand for Arctic offshore oil due to its lower carbon emissions during production compared to unconventional oil. The cumulative Arctic offshore oil production from all AUs by 2100 under LowC|2.6 is 213.6 EJ, slightly lower than the combined production under RCP 2.6 and LowC (110.5 EJ and 113.1 EJ, respectively). This effect reflects the increased competitiveness of Arctic offshore oil production as the technology costs decrease with thinning sea ice along with its lower emissions profile compared to unconventional oil.

The production quantity of Arctic offshore oil depends on the available oil resources and the sea-ice conditions within each AU. In some AUs, cumulative offshore oil production by 2100 reaches 70EJ, while others have none (Fig. 5). The quantity also varies across scenarios within regions. Most notably, the Alaska Platform cumulative production under Ref and LowC is only 5EJ but is approximately 60EJ under RCP 8.5, which has sea-ice conditions more favorable for lower-cost offshore oil extraction. This difference is due to the large oil resources in the Alaska Platform AU (Table S2), which leads to large increases in production when economically viable. All other AUs are associated with a cumulative production through 2100 of 20EJ or less under all scenarios.

In the near term (before 2030), none of the currently non-producing AUs begins production under any scenario. The expansion of Arctic offshore oil production occurs mid-century and beyond, as viable technology and demands change under the different scenarios.

Different from oil, Arctic offshore gas production is projected to occur at two AUs with the lowest sea-ice thickness levels (Barents Platform South and Arctic Norwegian Sea) under all scenarios (Fig. S25). The former AU has positive historical production levels, and the latter is projected to start production around 2020 with a relatively large cumulative amount that accrues within a shorter time frame.

### Effects on global oil and gas markets

**Figure 6:**
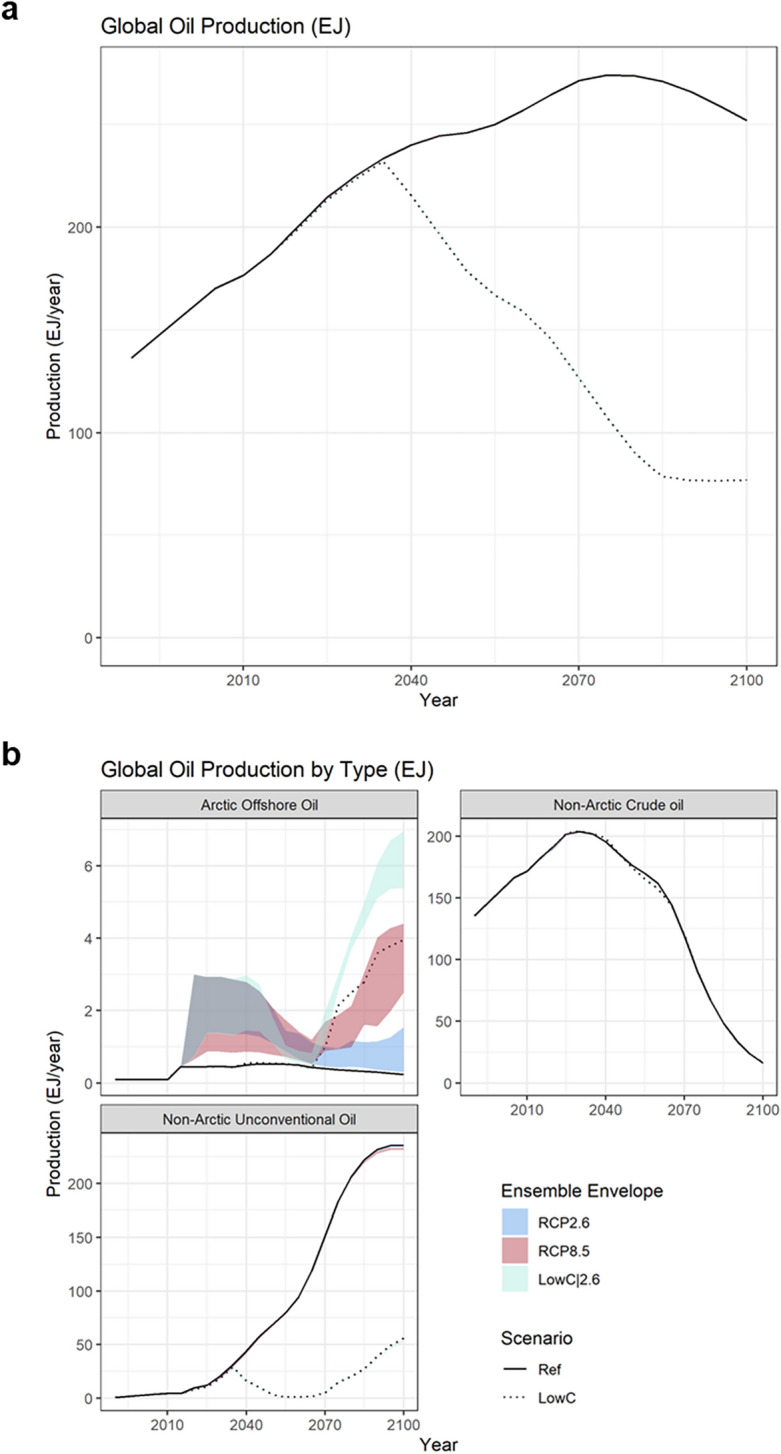
(**a**) Global oil production as a sum of Arctic offshore oil (crude/conventional oil), non-Arctic crude oil, and non-Arctic unconventional oil over time under model scenarios; (**b**) Global oil production by the three types of oil over time under the same model scenarios. Note that the differences between Ref, RCP2.6, and RCP 8.5 are negligible in (**a**), as well as in (**b**) for non-Arctic oils. Additionally, the differences among all scenarios are negligible for non-Arctic crude oil production in (**b**). The exajoule (EJ) is equal to 10^18^ J and approximately 0.2 billion barrels of oil equivalent (BBOE).

Under RCP2.6 and 8.5, depending on the sea-ice conditions, future extraction of Arctic offshore oil and gas contributes 1.3–2.6 EJ/year on average over 2020 to 2100 to oil and gas markets, compared to 0.8 EJ/year under Ref. However, this is not likely to affect the global oil and gas market through 2100. As shown, total oil production (a sum of Arctic offshore oil, non-Arctic crude, and unconventional oil production) are unaffected under RCP2.6 and 8.5 over time, compared to Ref (Fig. 6a), similarly for gas (Fig. S26). Similarly, compared to LowC, total oil and gas production under LowC|2.6 are unaffected (Fig. 6a and Fig. S26), although future Arctic offshore oil and gas extraction under LowC|2.6 adds 2.7–3.6 EJ/year compared to 1.8 EJ/year under LowC. The main reason is that the resource potential of Arctic offshore oil and gas is an insignificant portion of the world’s total remaining discovered and undiscovered oil and gas resource pool (Fig. S27). Moreover, the extraction of Arctic offshore oil and gas is relatively expensive compared to its competing resources, even under thinning sea-ice conditions.

Compared to Ref, LowC leads to lower global production of both oil and gas (Fig. 6a and Fig. S26). For oil, the impact of GHG emission reductions mainly acts on the “dirtier” unconventional oil production with zero production projected around the mid-century, although depletion of non-Arctic crude (conventional) oil towards the end of the century raises the production of unconventional oil as well as the Arctic offshore oil to meet the overall oil demand (Fig. 6b) with increasing oil prices (Fig. S28). The effect on natural gas prices is smaller than oil prices with generally lower natural gas prices under LowC than Ref (Fig. S29).

## Conclusion and discussions

Our results suggest that climate change impacts on Arctic sea-ice thickness will increase Arctic offshore oil production, though with that impact alone, it is likely to remain a small portion of total global oil production. The climate impacts on Arctic sea ice on offshore natural gas extraction are likely to be limited, with production remaining low due to the very low relative costs of onshore natural gas extraction elsewhere in the world. Among Arctic regions, changes in sea ice thickness are likely to have the largest impacts on USA Arctic offshore oil production, particularly for the Alaskan Platform AU. We find the effects of human efforts to achieve a low-carbon future (LowC scenarios) would *increase* Arctic offshore oil production, due to the simultaneous effect of reduction in unconventional oil production and the gradual depletion of non-Arctic crude oil over the century. Nevertheless, in the near term (before 2030), neither the effects of climate change-induced sea ice thinning nor low-carbon transition scenarios are likely to cause expansion of Arctic offshore oil production into new AUs. In addition, under the explored scenarios, future Arctic offshore oil and gas extraction is not likely to affect prices or production in the broader global oil and gas markets.

We explore the two-way interactions between energy market forces and climate change (including the consideration of human efforts to reach a low-carbon future) on the relative attractiveness of exploiting those resources. We nonetheless note that those two drivers are hardly the only forces at work in shaping oil and gas production in the region and that the final determination of how much the region’s fossil fuel resources will be exploited will depend on the sum of all the forces operating in the region. These scenarios are not intended to be a full exploration of how future energy technologies will unfold, but rather to provide useful information as to how the changing climate will alter accessibility to and the attractiveness of offshore oil and gas in the Arctic.

Other key forces or factors shaping the future Arctic offshore oil and gas extraction include technology barriers and advancements, public policies, environmental and safety concerns, and other externalities related to Arctic offshore extraction. In this section, we provide detailed discussions of each factor below, followed by limitations of this study including uncertainties in resource potential estimates and the lack of explicit modeling for managing associated gas and transportation costs.

Technology plays a pivotal role here. First, not all Arctic nations possess the modern technology and expertise to conduct offshore drilling in the Arctic, where unique challenges present due to the harsh environmental conditions. Consequently, countries lacking the necessary technology would need to rely on importing these capabilities from foreign nations or collaborating with international oil companies, contingent upon international relationships. This can greatly affect the feasibility of extracting offshore oil and gas resources from the Arctic. For example, Russia’s drilling operation in the Kara Sea in 2014 revealed oil resources of more than 130 million tons; however, a following drilling scheduled for 2015 was halted as a result of Western sanctions, which restricted Russia’s access to necessary offshore technology^13,14^. More recently, the European Union’s ban on most imports of Russian oil in addition to the restrictions of technology export to Russia^15^ may also have implications for Russia’s strategies regarding the expansion of its activities in the Arctic region.

Additionally, as technology continues to evolve for alternative energy sources, such as solar, wind, and nuclear power, their competitiveness could potentially replace or reduce the demand for fossil fuels. With a capped demand for fossil fuels, technological innovations in extracting non-Arctic unconventional oil could also lower the attractiveness of Arctic offshore oil. Although changes in technology improvement rates are defined by business-as-usual assumptions in this study, providing a baseline from which we can evaluate the *relative* impacts of climate change on Arctic offshore oil and gas extraction, our model is readily applicable to explore a range of scenarios involving changes for various technologies.

In addition to technology changes, motivation to explore and develop fossil fuel resources in the Arctic will also depend on national and corporate responses to other drivers. Oil price fluctuations can be greatly affected by unpredictable events as we have seen from COVID-19, responses to Russia’s invasion of Ukraine, and evolving public policies and public opinions are also important determinants of the motivation for Arctic oil and gas exploration. The current climate crisis inevitably raises concerns on fossil fuel reliance, particularly regarding the extraction of oil and gas from the nearly untapped Arctic offshore areas. In Norway, Arctic offshore development has been a priority for the nation since 2005 to support its economy and to satisfy growing energy demand nationally and internationally^16^. Yet, a recent shift in domestic opinions on the nation’s further Arctic oil explorations has led Norwegian oil companies to increase commitments to renewable energy such as offshore wind^17^. Similarly, Greenland was searching for financial autonomy from the Danish central authorities by exploring Arctic offshore oil resources^18^, but abandoned these exploration plans in 2021 due to climate concerns^19^. In the case of Canada, its government has prohibited oil and gas work in offshore Canadian Arctic waters since 2019 due to environmental considerations^20^. Whether or not the Canadian government’s standpoint will change will significantly affect the nation’s future exploration in the offshore Arctic. In the United States, Chukchi Sea and Beaufort Sea planning areas in the offshore Arctic are currently withdrawn from oil and gas drilling^21–23^. While our results show increased Arctic offshore oil production in these U.S. Arctic offshore areas in future periods due to decreased sea ice thickness, these results represent the isolated impacts of climate change on production, without considering current regulations in our model. Moreover, within the overarching context of climate change and public opinions, oil companies are also concerned with their reputational risk. If potential Arctic explorations are widely disputed, their future decisions on Arctic oil and gas extraction would be affected.

In contrast, Russia inclines towards the utilization of its Arctic fossil resource potentials. Russia sees Arctic offshore oil and gas extraction as a strategic piece of its plan to develop its Northern regions, in order to secure its national interests. In addition to Arctic offshore oil and gas extraction, the plan also includes the development of infrastructure and the Northern Sea Route (NSR) along Russia’s northern coast^24^. However, given the unfavorable price trends of oil and gas since 2014 and restrictions on offshore technology, experts predict that large-scale offshore oil and gas production in the Russian Arctic will likely become feasible only after 2035^24,25^. This outlook also aligns with the latest Energy Strategy of the Russian Federation^26^, which declares significant growth in hydrocarbon production in the Arctic beyond 2035.

The Arctic’s unique environment also presents potential hazards that are uncommon elsewhere. Despite the decrease in sea-ice thickness, increasing access to offshore oil and gas, the expanding ice-free ocean will likely cause increases in wave heights^27,28^ and increases in the frequency and intensity of storms^29–31^. These hazards can endanger the safety of personnel and infrastructure in Arctic offshore extraction operations. Iceberg threats are another concern that could increase logistical challenges and operational costs. While these factors are likely incidental and their effects on the costs could vary case-by-case, future research could use our model to evaluate the “shock” of such events on prolonged increases in overall production costs and their implications for Arctic offshore oil and gas production.

Furthermore, the Arctic’s eco-sensitive nature amplifies the potential consequences of offshore oil and gas extraction activities. The risk of oil spills in these pristine waters stands as a looming concern, as the Arctic is particularly vulnerable to oil spills due to factors such as its fragile ecosystems and slow ecosystem recovery. Additionally, addressing oil spills in the remote and hazardous location is difficult and the current ability of Arctic nations to respond to such events is limited^32,33^. Conversely, there are also positive externalities stemming from drilling activities, such as job creation and infrastructure development, stimulating local economies. These multifaceted factors collectively shape decisions on Arctic exploration.

Our study aims to isolate the impacts of climate change and a transition to a low-carbon energy future on Arctic offshore oil and gas extraction, rather than give a definitive prediction of the future. This model can serve as a useful tool to explore additional factors and the associated outcomes of Arctic offshore oil and gas extraction as climate and non-climate conditions begin to resolve in the future. However, there are a few limitations of this study that could be improved in future research given available data.

First, the undiscovered Arctic offshore resources estimated by USGS are associated with uncertainties. In this study, we take the mean estimates to represent the current best guess of resource potential. The uncertainties explored in this study are based on uncertainties in sea-ice conditions under climate change, as projected by different global climate models. Future work could extend this to explore the effects of resource potential uncertainty on the outcomes or if an updated dataset on resource potential becomes available.

Second, we do not explicitly model the cost of managing the associated gas in oil production. Depending on the techniques and strategies in managing the associated gas, this industrial process can generate different costs and benefits, and impose different environmental impacts. For example, burning off associated gas at the wellsite, known as flaring, has been a common and simple practice with relatively low cost. However, regulations have been put in place to limit flaring due to its release of greenhouse gases and air pollutants in several countries, including two Arctic nations—Norway and the United States^34^. More sustainable management practices of associated gas in the future could lead to different costs and benefits in the overall oil production. This study assumes the non-explicitly modeled costs are consistent with a business-as-usual scenario; however, the model can be used to explore a range of scenarios such as an increasing of the overall oil production costs due to future changes in managing associated gas, which is not the focus of this study but can be of interest in future research.

Similarly, we do not explicitly model the costs of transporting the oil produced offshore to the demanded destinations, which can vary depending on the transportation modes (e.g., a combination of tankers and pipelines) and the proximity to the destinations (e.g., onshore processing sites, refining facilities, or direct-exporting destinations). Notably, sea-ice loss in the Arctic has made navigation easier in the region, particularly along the Northern Sea Route (NSR) off Russia’s northern coast. If the offshore oil produced needs to be shipped across the Arctic through the NSR, shipping costs are likely to be lower in the future with sea-ice loss under climate change. Future work may involve improving the representation of oil and gas transportation in the model.

While we provide valuable insights into the potential impacts of climate change and energy transitions on Arctic offshore oil and gas production and its implications for broader energy markets, it is essential to recognize the scope and limitations of our study. As Arctic conditions continue to evolve, future research can build on our study to advance understanding and modeling of Arctic offshore oil and gas production.

### Supplementary Information

Supplementary Information.

## Data Availability

The data and model implemented in this study are available at 10.5281/zenodo.10126664.
