# Supplementary material for: Limited increases in Arctic offshore oil and gas production with climate change and the implications for energy markets
Source: Sci Rep. 2024 Mar 20;14:6699. doi: 10.1038/s41598-024-54007-x (PMC10954641; doi:10.1038/s41598-024-54007-x)
Supplement: Supplementary file 1 — Supplementary Information. [file 41598_2024_54007_MOESM1_ESM.pdf]

Ambio

Supplementary Information

This supplementary information has not been peer reviewed.

Title: **Limited increases in Arctic offshore oil and gas production with climate change and the implications for energy markets**

Table S1: Selected climate models in CMIP6 for sea ice thickness data and subsequent analysis in this study.

**Model name**	**Number of Ensembles**
EC-Earth3	3
EC-Earth3-Veg	3
IPSL-CM6A-LR	3
MIROC6	3
MRI-ESM2-0	1

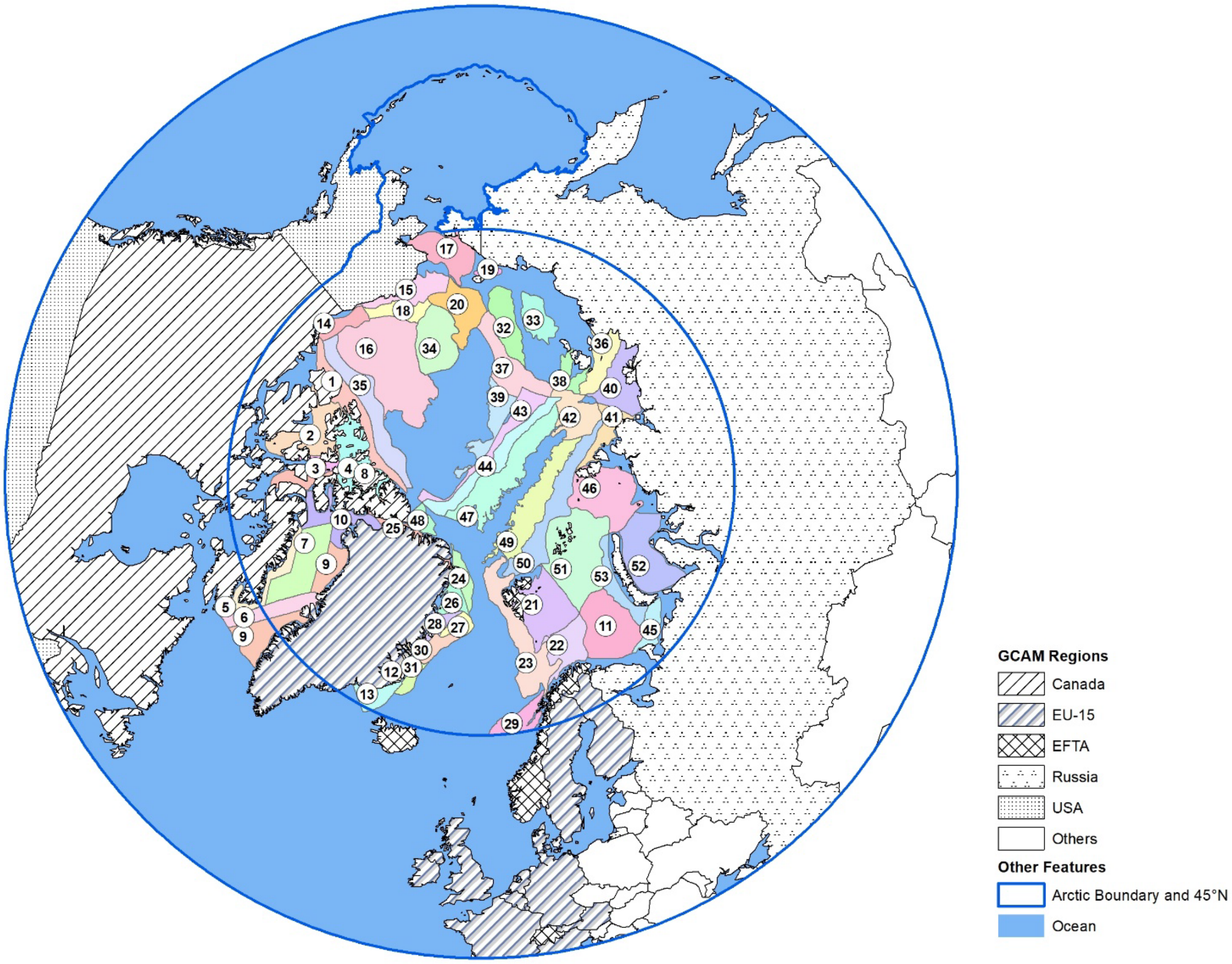

1 Banks Island-Sverdrup Rim	19 Long Strait	37 Siberian Passive Margin
2 Western Franklinian Shelf	20 North Chukchi-Wrangel Foreland	38 Anisin-Novosibirsk Basins
3 Boothia-Cornwallis Uplift	21 Barents Platform North	39 Podvodnikov-Makarov Basins
4 Sverdrup Upper Paleozoic	22 Barents Platform South	40 West Laptev Grabens
5 Northeast Canada Rifted Margin	23 Western Barents Margin	41 Northwest Laptev Sea Shelf
6 Greater Ungava Fault Zone	24 Wandel Sea Basin	42 Lena Prodelta
7 Baffin Bay Basin	25 Eastern Franklinian Shelf	43 Makarov Basin Margin
8 Sverdrup Mesozoic	26 North Danmarkshavn Salt Basin	44 Lomonosov Ridge
9 Northwest Greenland Rifted Margin	27 Thetis Basin	45 Kolguyev Terrace
10 Eurekan Structures	28 South Danmarkshavn Basin	46 North Kara Basins and Platforms
11 South Barents Basin and Ludlov Saddle	29 Arctic Norwegian Sea	47 Amundsen Basin
12 Jameson Land Basin	30 Northeast Greenland Volcanic Province	48 Lincoln Sea Basin
13 Jameson Land Basin Subvolcanic Extension	31 Liverpool Land Basin	49 Nansen Basin
14 Canning-Mackenzie Deformed Margin	32 Vilkitskii Basin	50 Nansen Basin Margin
15 Alaskan Platform	33 East Siberian Sea Basin	51 North Barents Basin
16 Canada Basin	34 Chukchi Borderland	52 South Kara Sea Offshore
17 Hope Basin	35 Canada Passive Margin	53 Novaya Zemlya Basins and Admiralty Arch
18 Alaska Passive Margin	36 East Laptev Horsts	

Fig. S1 Assessment Units (AUs) and GCAM regions. An index table for AUs is included. GCAM region definitions are shown in Table S3. We only include Assessment Units (AU) that are completely or almost

completely offshore, because USGS does not provide a detailed split of onshore and offshore resource potential estimation for AUs that are partially onshore or offshore, and we are interested in the sea ice loss impacts on Arctic offshore oil and gas production.

Table S2: Undiscovered oil and gas potential in offshore Assessment Units (AUs), calculated based on USGS estimates (Methods).

GCAM Region	Assessment Unit	Oil Potential (EJ)	Gas Potential (EJ)	AU index in Fig. S1
Canada	Baffin Bay Basin	4.44	6.47	7
Canada	Banks Island-Sverdrup Rim	2.42	3.84	1
Canada	Boothia-Cornwallis Uplift	NO DATA	NO DATA	3
Canada	Canada Basin	NO DATA	NO DATA	16
Canada	Canada Passive Margin	13.54	15.93	35
Canada	Canning-Mackenzie Deformed Margin	18.22	18.94	14
Canada	Eastern Franklinian Shelf	NO DATA	NO DATA	25
Canada	Eurekan Structures	3.24	4.53	10
Canada	Greater Ungava Fault Zone	4.78	7.13	6
Canada	Lincoln Sea Basin	2.48	3.46	48
Canada	Northeast Canada Rifted Margin	8.18	9.17	5
Canada	Sverdrup Mesozoic	2.44	5.22	8
Canada	Sverdrup Upper Paleozoic	NO DATA	NO DATA	4
Canada	Western Franklinian Shelf	NO DATA	NO DATA	2
Greenland	Baffin Bay Basin	4.44	6.47	7
Greenland	Eastern Franklinian Shelf	NO DATA	NO DATA	25
Greenland	Eurekan Structures	3.24	4.53	10
Greenland	Greater Ungava Fault Zone	4.78	7.13	6
Greenland	Jameson Land Basin	NO DATA	NO DATA	12
Greenland	Jameson Land Basin Subvolcanic Extension	NO DATA	NO DATA	13
Greenland	Lincoln Sea Basin	2.48	3.46	48
Greenland	Liverpool Land Basin	1.20	1.81	31
Greenland	North Danmarkshavn Salt Basin	18.70	42.21	26
Greenland	Northeast Greenland Volcanic Province	2.84	4.33	30
Greenland	Northwest Greenland Rifted Margin	28.01	33.53	9
Greenland	South Danmarkshavn Basin	25.04	37.93	28
Greenland	Thetis Basin	3.07	4.63	27
Greenland	Wandel Sea Basin	2.76	3.85	24
Norway	Arctic Norwegian Sea	7.01	28.54	29
Norway	Barents Platform North	2.11	6.41	21
Norway	Barents Platform South	9.63	21.25	22

Norway	Western Barents Margin	1.20	5.51	23
Russia	Amundsen Basin	NO DATA	NO DATA	47
Russia	Anisin-Novosibirsk Basins	2.68	3.66	38
Russia	East Laptev Horsts	NO DATA	NO DATA	36
Russia	East Siberian Sea Basin	0.11	0.65	33
Russia	Kolguyev Terrace	0.83	2.73	45
Russia	Lena Prodelta	5.59	16.36	42
Russia	Lomonosov Ridge	NO DATA	NO DATA	44
Russia	Long Strait	NO DATA	NO DATA	19
Russia	Makarov Basin Margin	0.70	0.99	43
Russia	Nansen Basin	NO DATA	NO DATA	49
Russia	Nansen Basin Margin	2.08	4.19	50
Russia	North Barents Basin	30.40	134.63	51
Russia	North Chukchi-Wrangel Foreland	0.49	6.40	20
Russia	North Kara Basins and Platforms	10.32	15.80	46
Russia	Northwest Laptev Sea Shelf	0.98	4.73	41
Russia	Novaya Zemlya Basins and Admiralty Arch	NO DATA	NO DATA	53
Russia	Podvodnikov-Makarov Basins	NO DATA	NO DATA	39
Russia	Siberian Passive Margin	5.62	6.56	37
Russia	South Barents Basin and Ludlov Saddle	11.08	197.66	11
Russia	South Kara Sea Offshore	14.32	656.44	52
Russia	Vilkitskii Basin	0.56	6.06	32
Russia	West Laptev Grabens	15.12	30.69	40
USA	Alaska Passive Margin	5.55	6.21	18
USA	Alaskan Platform	159.09	168.33	15
USA	Canada Basin	NO DATA	NO DATA	16
USA	Canning-Mackenzie Deformed Margin	18.22	18.94	14
USA	Chukchi Borderland	NO DATA	NO DATA	34
USA	Hope Basin	0.01	0.69	17

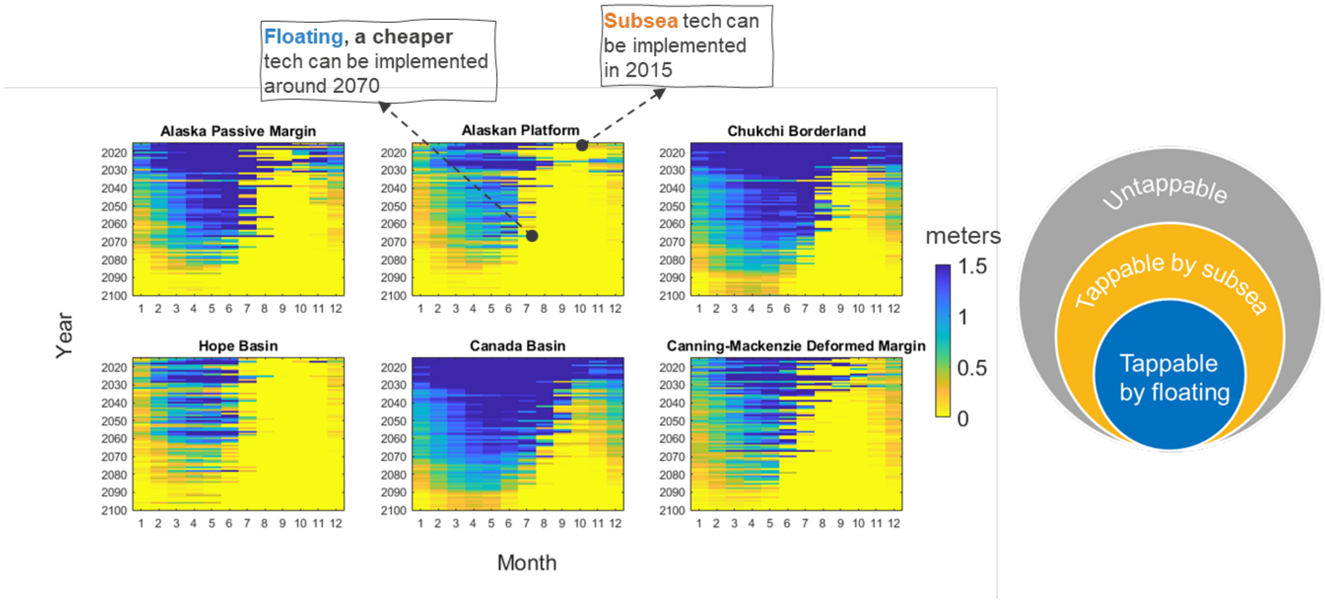

Fig. S2 An illustration of how to determine whether an Assessment Unit is tappable by different offshore extraction technologies. Overall, an AU in a year is either untappable, tappable only by subsea, or tappable by floating. The sea ice thickness data used in this illustration is from CMIP6 EC-Earth3-Veg Ensemble #1 under RCP8.5.

Appendix S1

Estimating Extraction Costs

The estimation of extraction costs of Arctic offshore oil and gas is based on a previous study which provides cost estimates for two different offshore technologies (Petrick et al. 2017) and other estimates from literature and reports (International Energy Agency 2008; Marszałkowski 2019). Although the data on such cost estimates are limited, we anchored the upper and lower limit of the costs from International Energy Agency (IEA) and several middle points of cost estimates as found in the literature. After that, we combine the cost estimates with annual average sea ice thickness (hereinafter "sea ice thickness") provided by CMIP6 (Eyring et al. 2016) in the model base year (i.e., 2015), to build a two-phase linear relationship between extraction costs and the sea ice thickness (Fig. S27 and S28).

Specifically, we assume that the upper bound of extraction cost applies to the more expensive offshore technology (i.e., subsea) and the lower bound applies to the less expensive offshore technology (i.e., floating). We then linearly extrapolate the points of extraction costs and associated sea ice thickness for each technology, until the costs of two technologies meet at the mean sea ice thickness of AUs that are tappable by both technologies. We then calculate the percent difference of the two technologies' costs. By assuming that the extraction cost of floating is always lower than the cost of subsea at such percent difference across different sea ice thicknesses, we obtain the two-phase cost function for each offshore technology, and for extracting oil and gas, respectively (Fig. S28).

The derived cost functions indicate that the extraction costs increase rapidly as sea ice thickness increases from zero to the mean sea ice thickness of AUs that are tappable by both technologies, after which the costs increase relatively slowly. As a result, the derived costs functions for tappable AUs are:

$$Cost^{oil,\,subsea} = \begin{cases} 4.61 * SIT + 1.71 & when\ 0 < SIT \leq 0.32\ m \\ 0.38 * SIT + 3.06 & when\ SIT > 0.32\ m \end{cases}$$

$$Cost^{oil,\,floating} = \begin{cases} 4.14 * SIT + 1.53 & when\ 0 < SIT \leq 0.32\ m \\ 0.34 * SIT + 2.75 & when\ SIT > 0.32\ m \end{cases}$$

$$Cost^{gas,\,subsea} = \begin{cases} 3.50 * SIT + 1.55 & when\ 0 < SIT \leq 0.32\ m \\ 0.55 * SIT + 2.49 & when\ SIT > 0.32\ m \end{cases}$$

$$Cost^{gas,\,floating} = \begin{cases} 3.46 * SIT + 1.53 & when\ 0 < SIT \leq 0.32\ m \\ 0.54 * SIT + 2.46 & when\ SIT > 0.32\ m \end{cases}$$

where SIT is the annual average sea ice thickness at an AU.

When an AU is tappable given the monthly sea ice thickness criteria (described in the main text), the extraction costs are calculated using the derived functions above. When an AU is not tappable, the costs are assumed to be a large enough number such that the model would not consider extracting the resource at this AU (Fig. S29). As a result, annual extraction costs for oil and gas, respectively, are estimated over 2015-2100, for each AU, each climate ensemble, and under each RCP scenario.

a. Oil

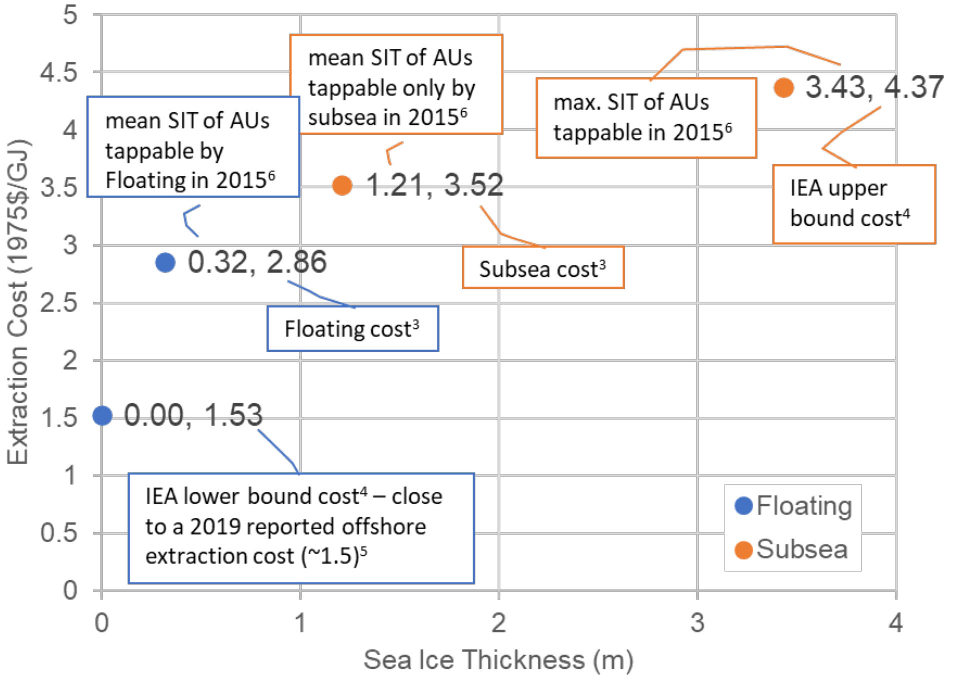

b. Gas

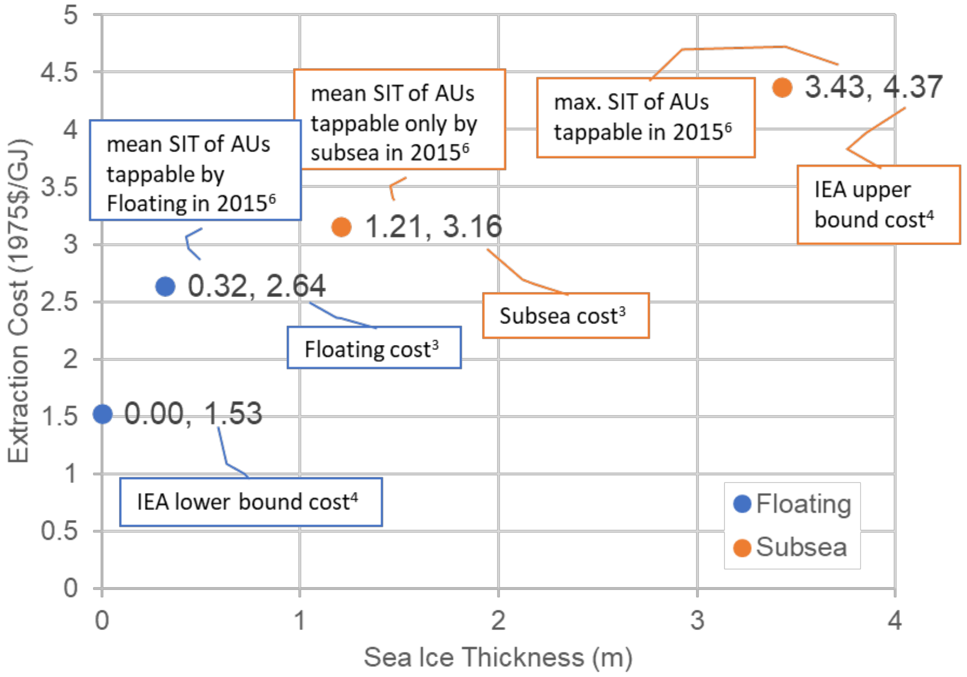

Fig. S3 Data of extraction cost of Arctic offshore oil (gas) and sea ice thickness used for designing cost functions using two different offshore extraction technologies – floating and subsea. Floating is a cheaper technology than subsea as described in a detailed study (Petrick et al. 2017). SIT – Sea Ice Thickness. AUs – Assessment Units. IEA – International Energy Agency. The values are cited accordingly.

a. Oil

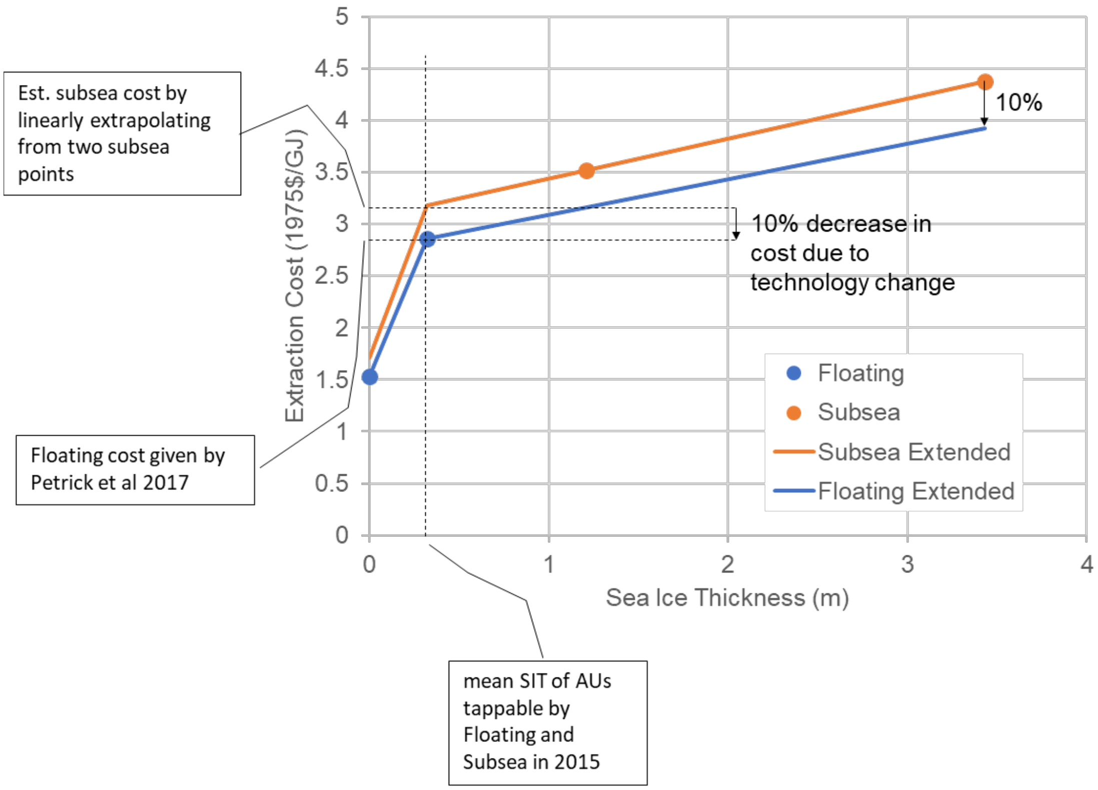

b. Gas

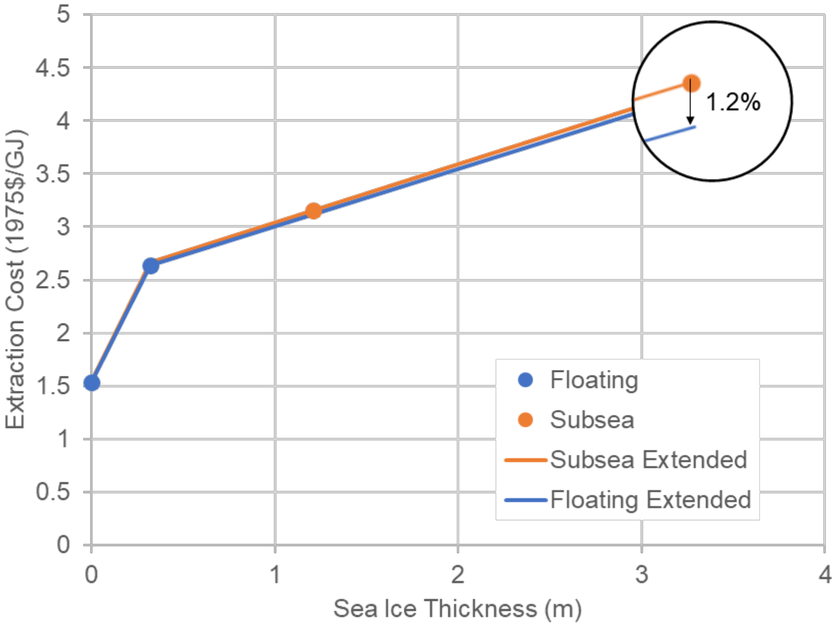

Fig. S4 Derived cost functions for Arctic offshore oil extraction using two different offshore technologies (i.e., floating and subsea). Note that for Arctic offshore oil, the extraction cost using floating technology is always 10% lower than the subsea technology. For Arctic offshore gas, the percent difference is lower at 1.2%.

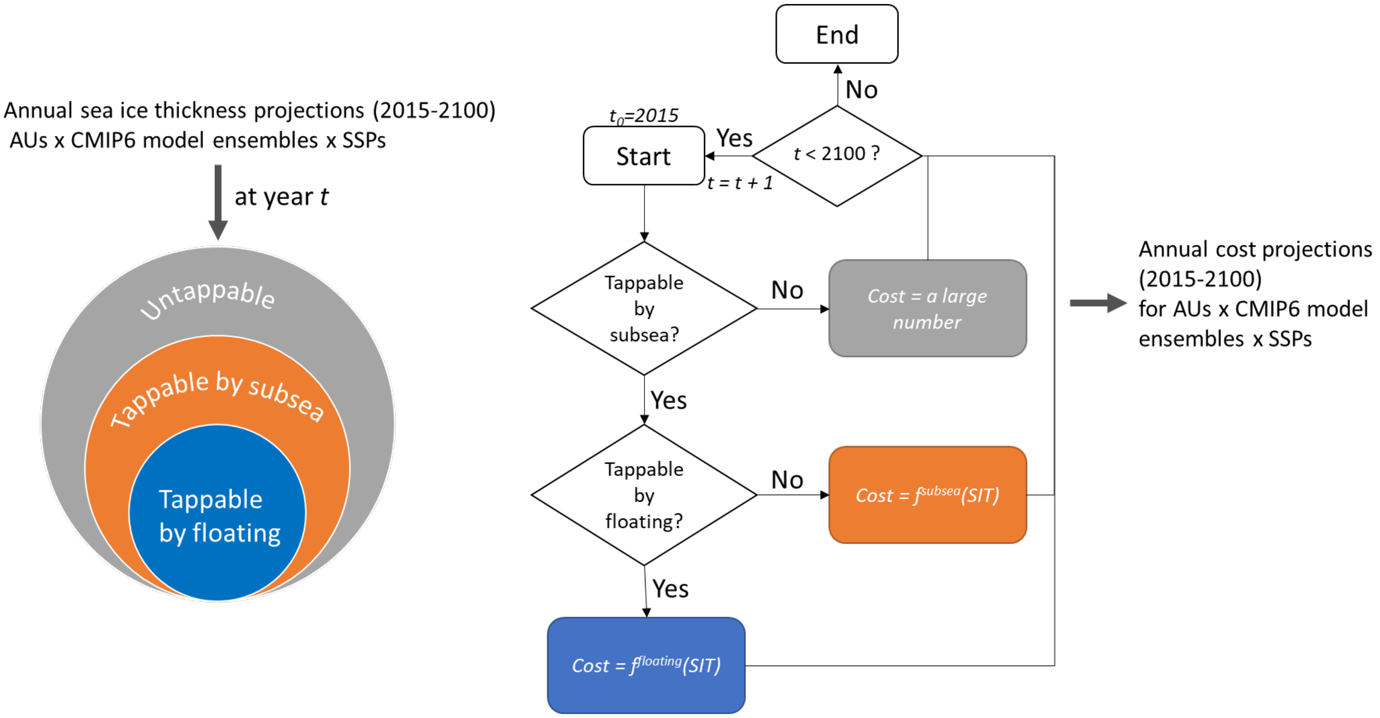

Fig. S5 Process of estimating extraction costs at each AU and time step, cross CMIP6 model ensembles of sea ice thickness projections under different SSPs.

Table S3: Mapping from key GCAM regions in this study to country

**GCAM Region**	**Countries**
Canada	Canada
EU-15	Andorra, Austria, Belgium, Denmark, Finland, France, Germany, Greece, **Greenland**, Ireland, Italy, Luxembourg, Monaco, Netherlands, Portugal, Sweden, Spain, United Kingdom
European Free Trade Association (EFTA)	Iceland, **Norway**, Switzerland
Russia	Russia
USA	United States

a. Oil

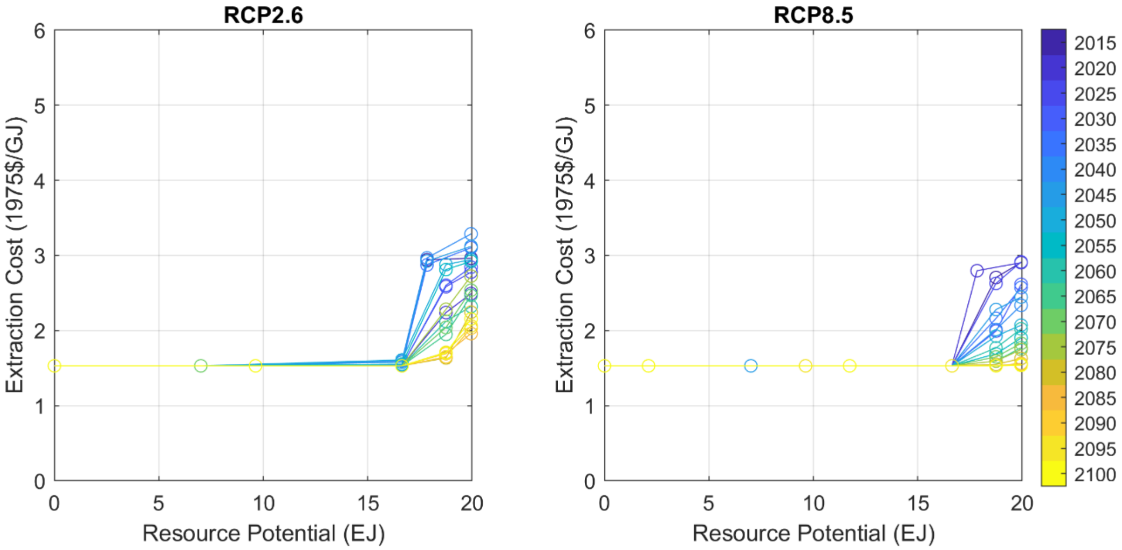

b. Gas

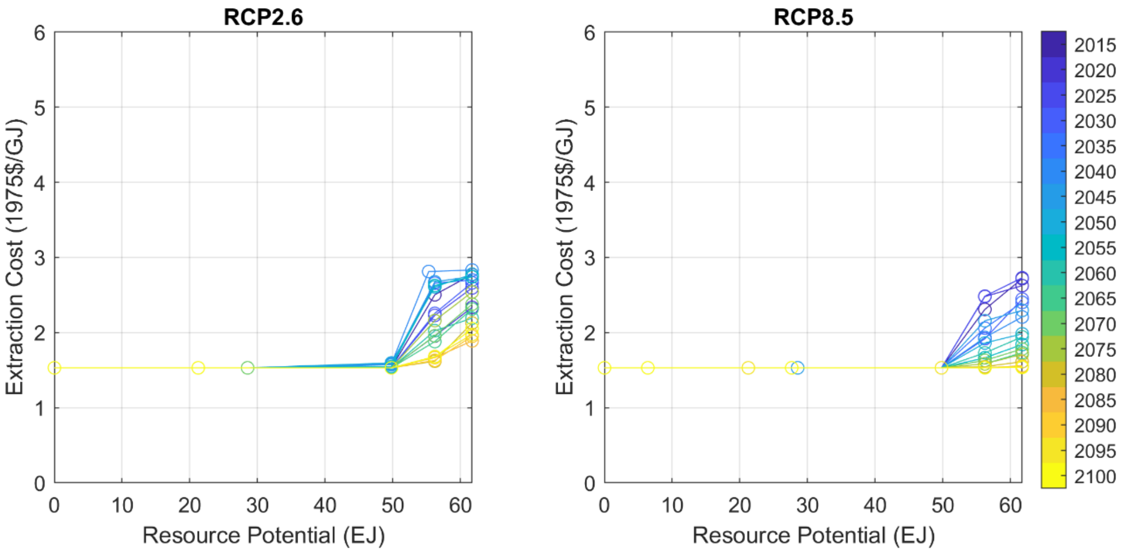

Fig. S6 Resource supply curves for Arctic offshore **a**) oil and **b**) gas in **Norway** under RCP2.6 and 8.5. For illustration purposes, resource supply curves based on only one climate ensemble (i.e., CMIP6 EC-Earth3-Veg Ensemble #1) is shown here.

a. Oil

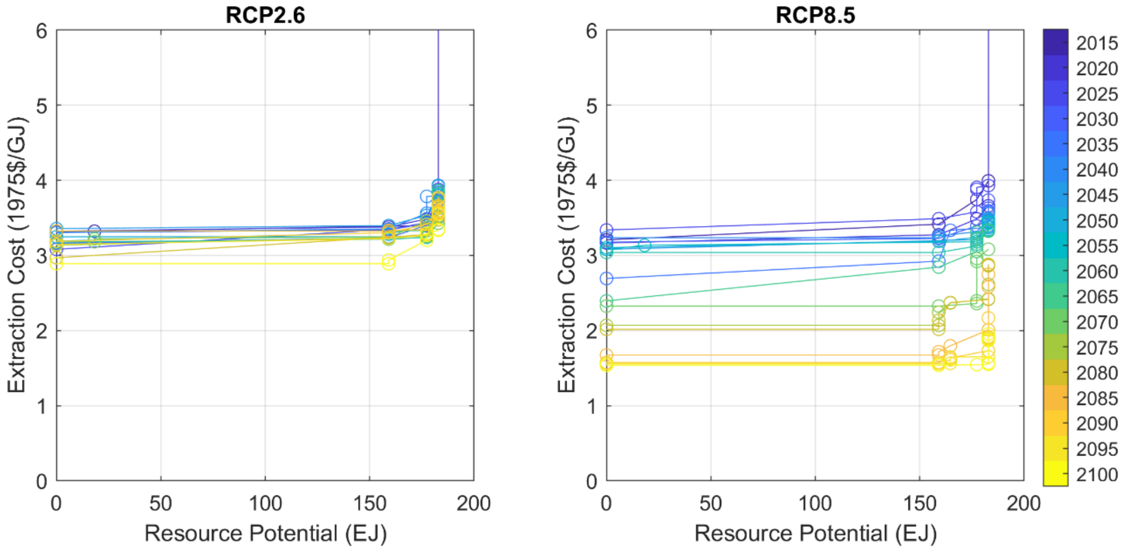

b. Gas

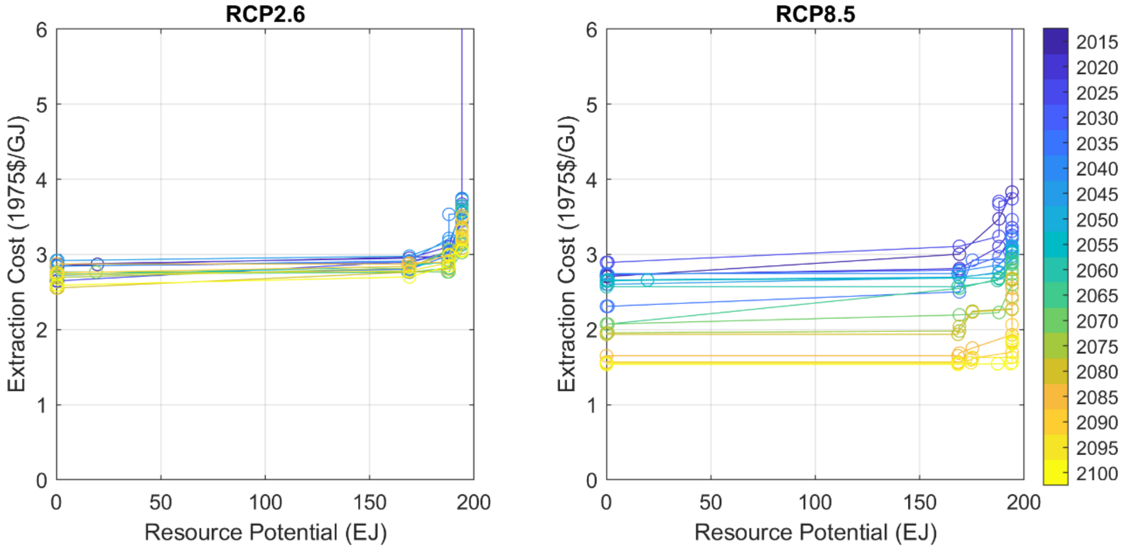

Fig. S7 Resource supply curves for Arctic offshore **a**) oil and **b**) gas in the **USA** under RCP2.6 and 8.5. For illustration purposes, resource supply curves based on only one climate ensemble (i.e., CMIP6 EC-Earth3-Veg Ensemble #1) is shown here.

a. Oil

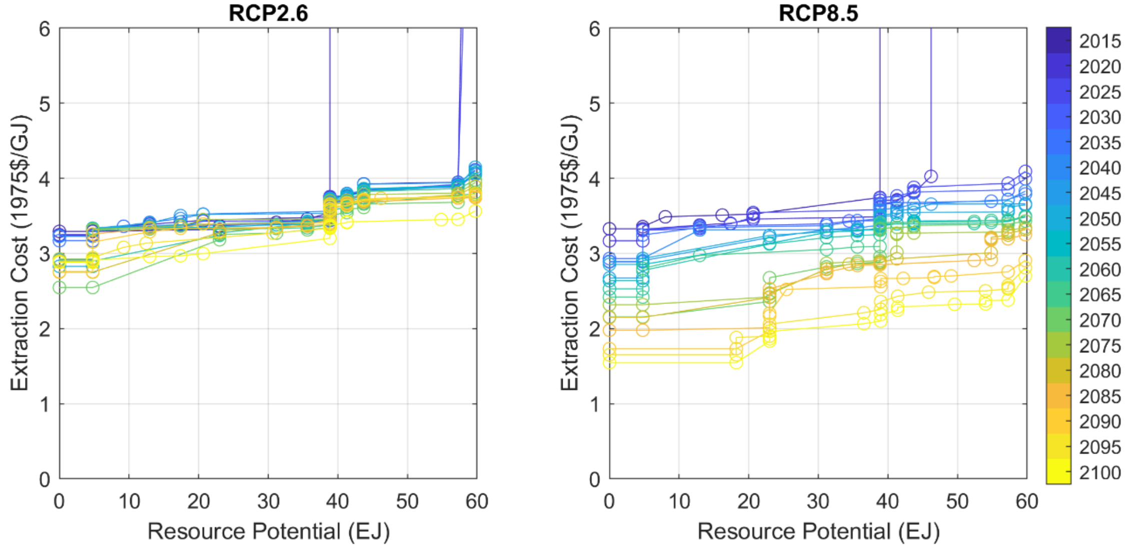

b. Gas

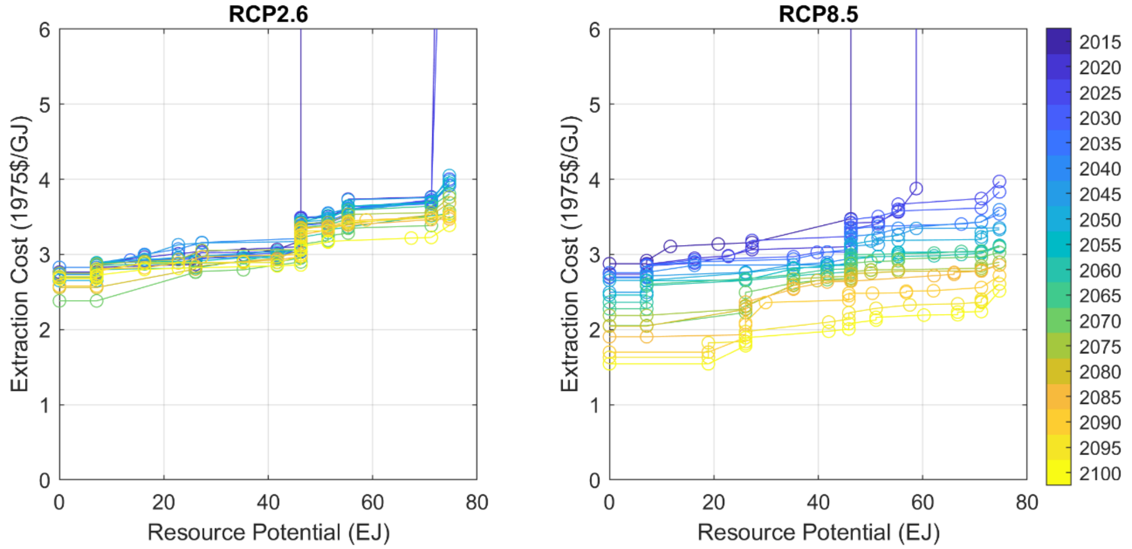

Fig. S8 Resource supply curves for Arctic offshore **a**) oil and **b**) gas in **Canada** under RCP2.6 and 8.5. For illustration purposes, resource supply curves based on only one climate ensemble (i.e., CMIP6 EC-Earth3-Veg Ensemble #1) is shown here.

a. Oil

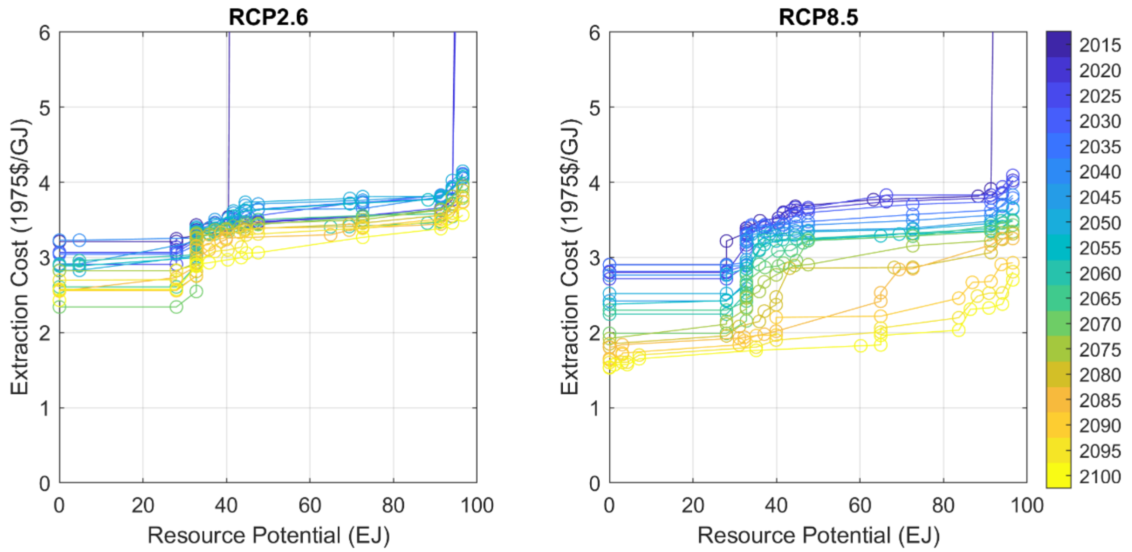

b. Gas

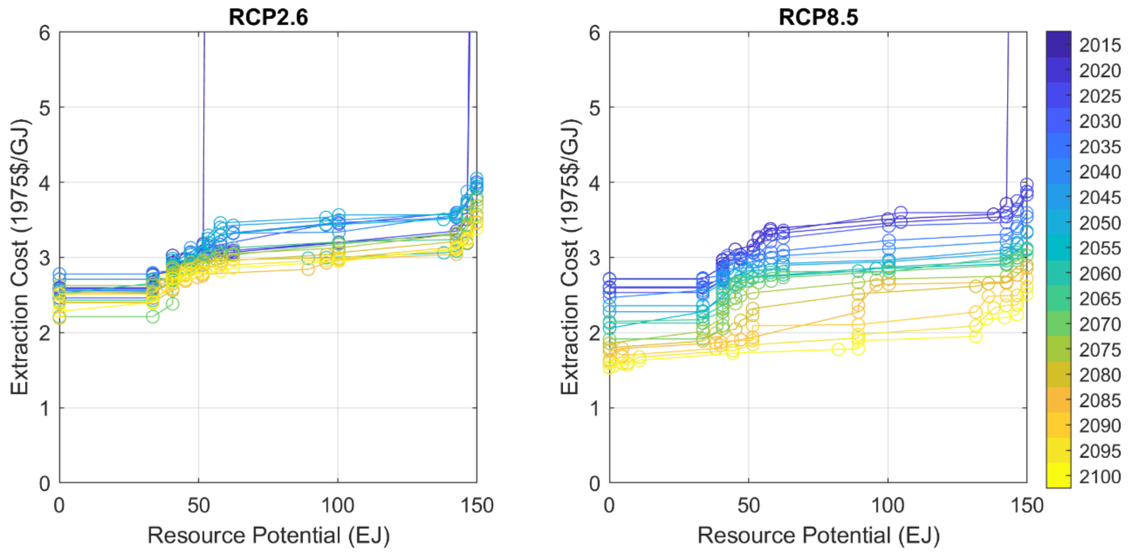

Fig. S9 Resource supply curves for Arctic offshore **a**) oil and **b**) gas in **Greenland** under RCP2.6 and 8.5. For illustration purposes, resource supply curves based on only one climate ensemble (i.e., CMIP6 EC-Earth3-Veg Ensemble #1) is shown here.

a. Oil

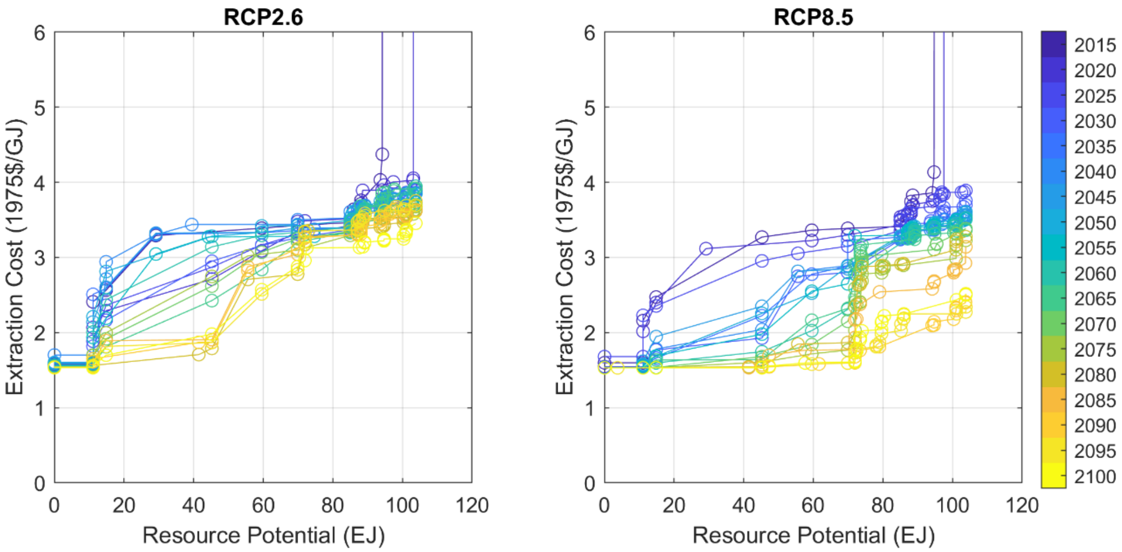

b. Gas

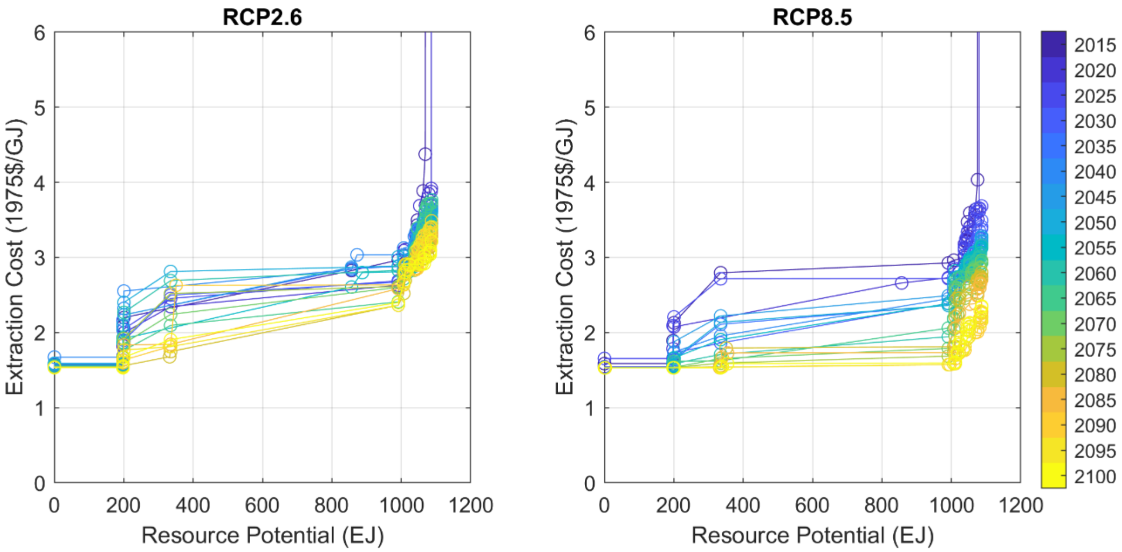

Fig. S10 Resource supply curves for Arctic offshore **a**) oil and **b**) gas in **Russia** under RCP2.6 and 8.5. For illustration purposes, resource supply curves based on only one climate ensemble (i.e., CMIP6 EC-Earth3-Veg Ensemble #1) is shown here.

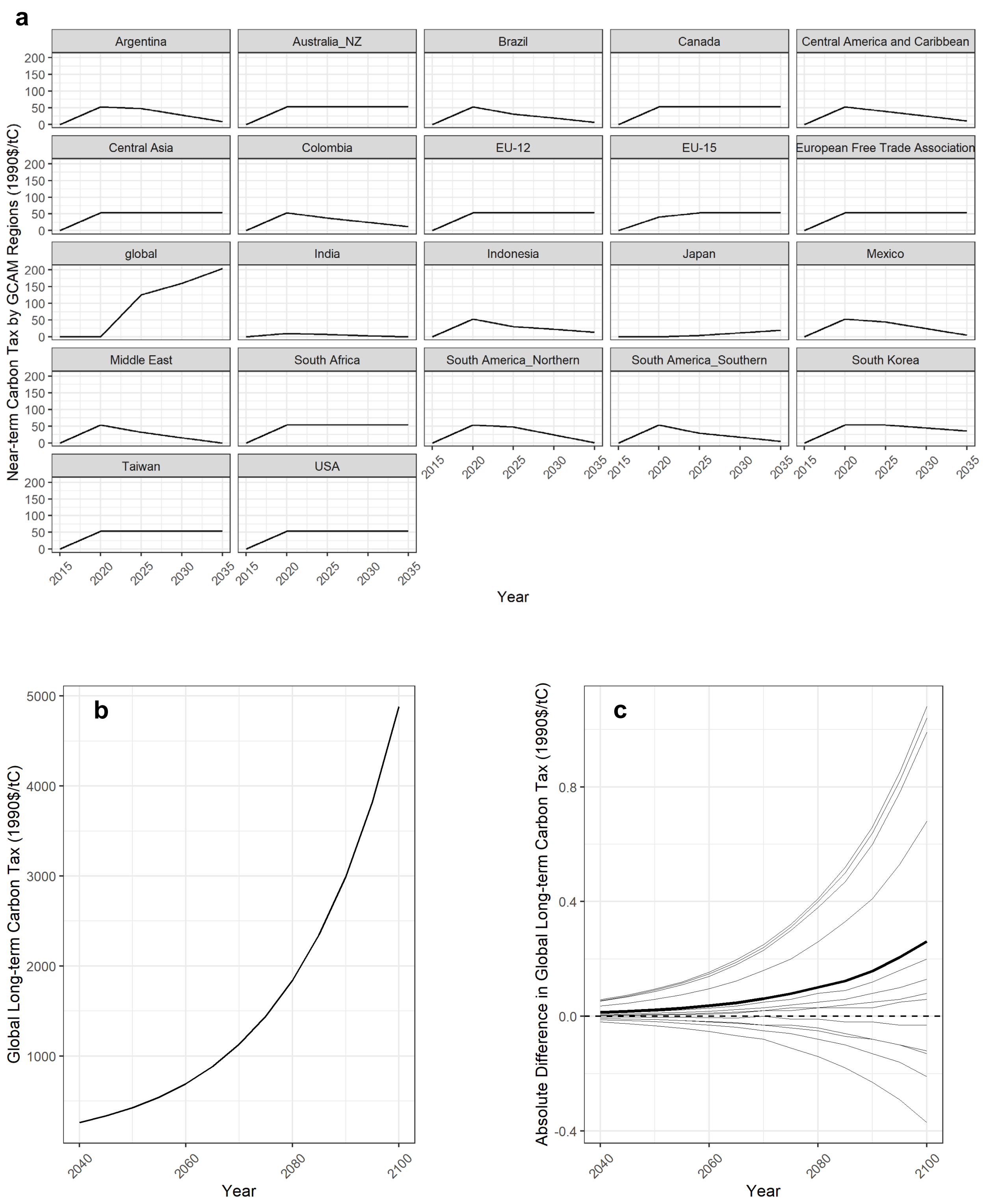

Fig. S11 Carbon tax pathway under LowC **a**) in the near term (through 2035) by region and **b**) in the long term (2040-2100) globally. The near-term carbon tax pathway under LowC|2.6 is the same as LowC. For the long term, the absolute differences of carbon tax under LowC|2.6 compared to LowC are shown in **c**), where thinner lines are for LowC|2.6 ensembles and the thicker line is the ensemble average. Note that regions with zero carbon tax over the entire near-term period are not shown in **a**).

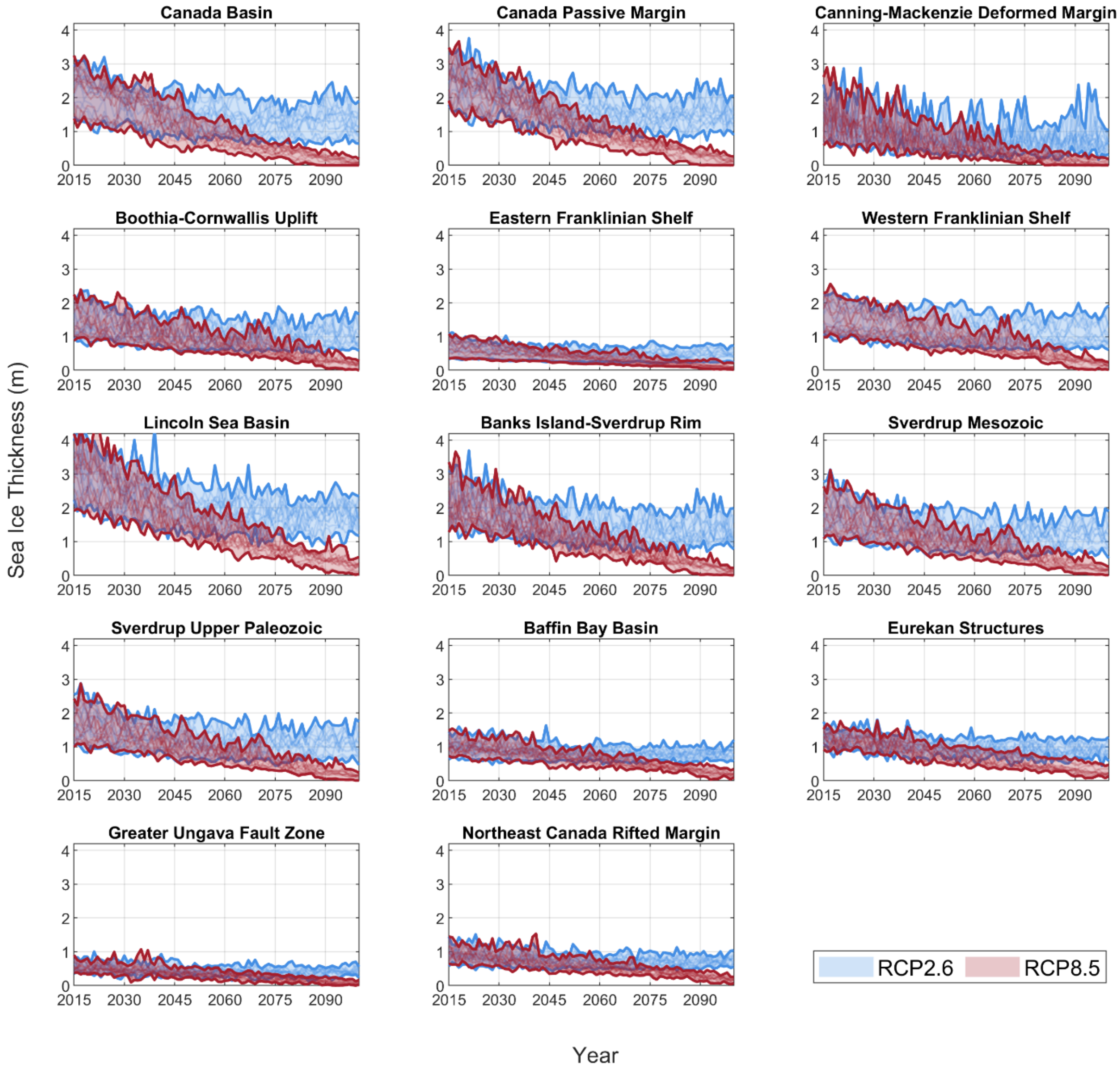

Fig. S12 Annual sea ice thickness over 2015-2100 across Assessment Units (AUs) in **Canada**, based on projections from CMIP6 model ensembles under RCP2.6 and 8.5 (Methods). Each RCP category is shown in a single-color band bounded by the maximum and minimum value of all ensembles over years.

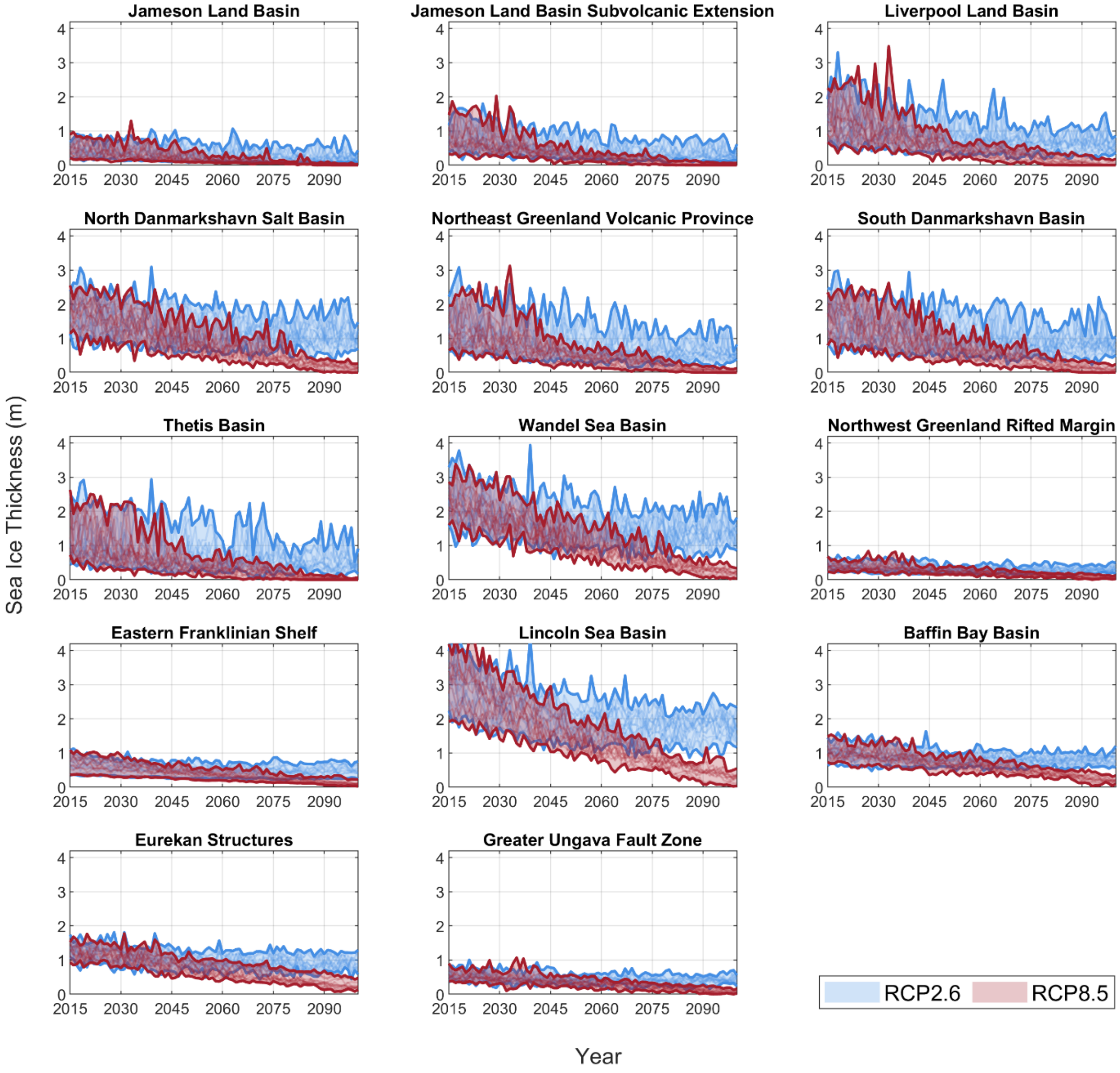

Fig. S13 Annual sea ice thickness over 2015-2100 across Assessment Units (AUs) in **Greenland** (part of EU-15), based on projections from CMIP6 model ensembles under RCP2.6 and 8.5 (Methods). Each RCP category is shown in a single-color band bounded by the maximum and minimum value of all ensembles over years.

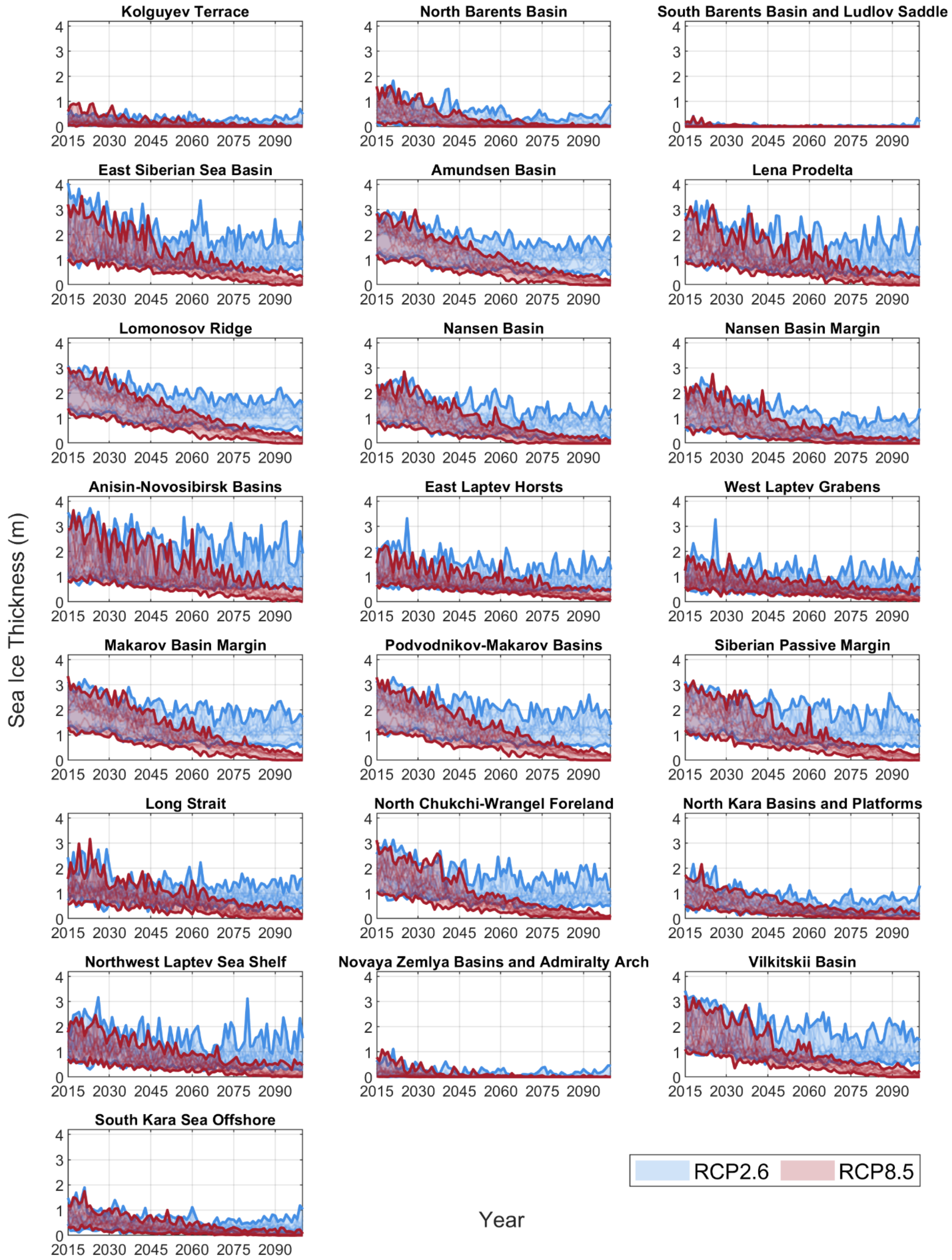

Fig. S14 Annual sea ice thickness over 2015-2100 across Assessment Units (AUs) in **Russia**, based on projections from CMIP6 model ensembles under RCP2.6 and 8.5 (Methods). Each RCP category is shown in a single-color band bounded by the maximum and minimum value of all ensembles over years.

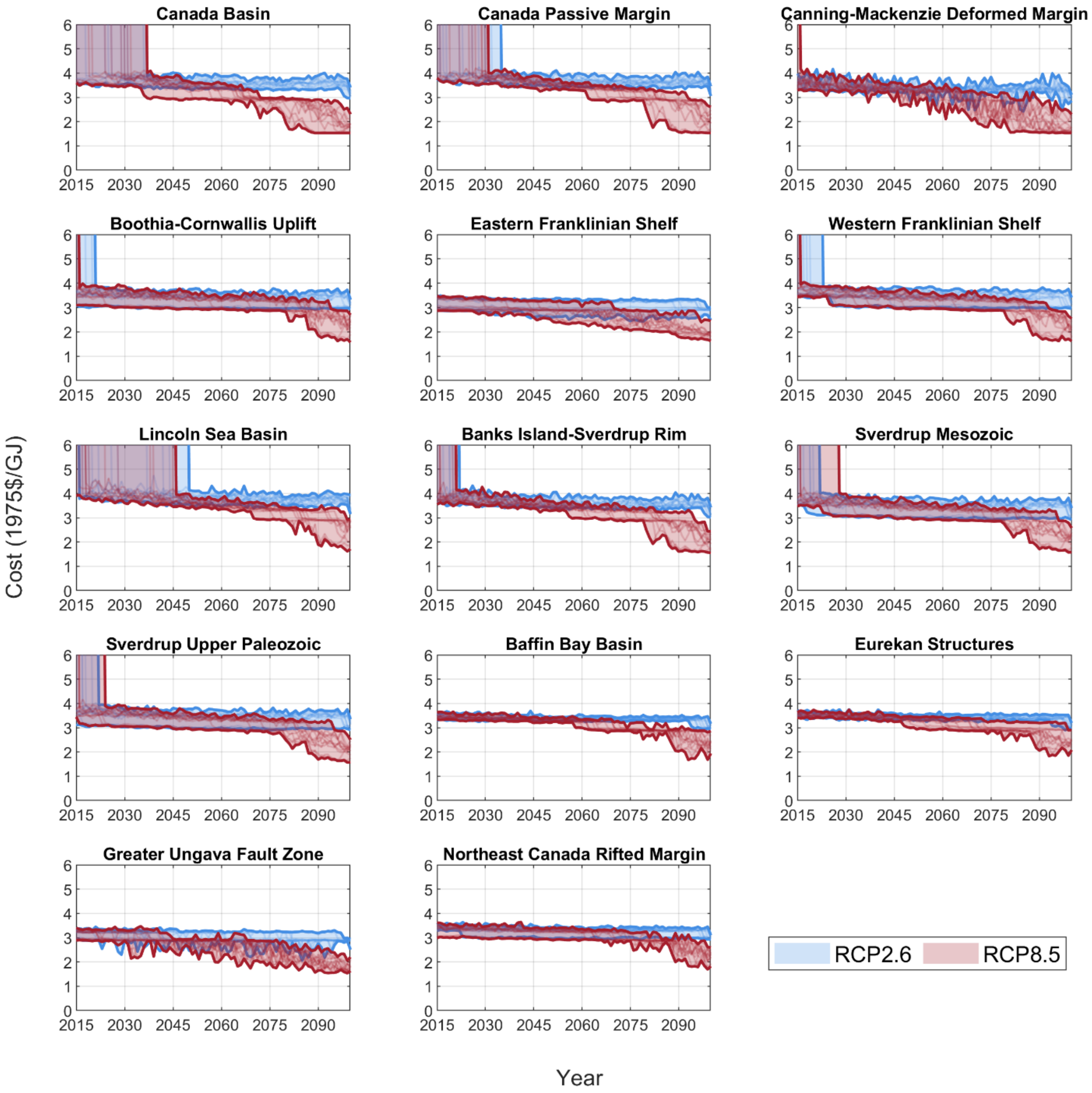

Fig. S15 Extraction costs for Arctic offshore *oil* over 2015-2100 across Assessment Units (AUs) in **Canada**, given sea ice thickness projections from CMIP6 model ensembles under RCP2.6 and 8.5. Each RCP category is shown in a single-color band bounded by the maximum and minimum value of all ensembles over years. Costs beyond the y-axis upper limit indicate that the assessment unit is untappable at that time.

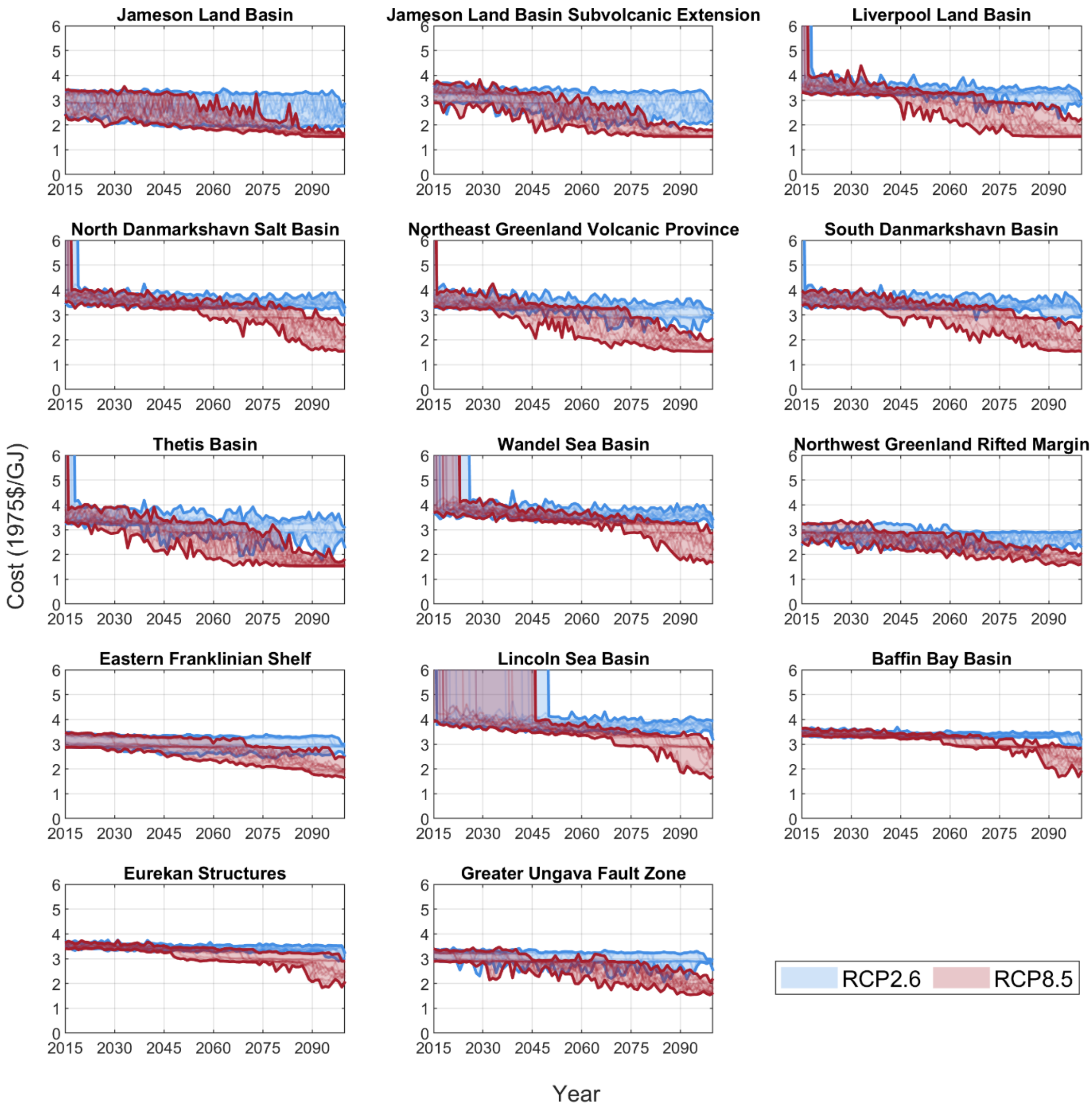

Fig. S16 Extraction costs for Arctic offshore *oil* over 2015-2100 across Assessment Units (AUs) in **Greenland**, given sea ice thickness projections from CMIP6 model ensembles under RCP2.6 and 8.5. Each RCP category is shown in a single-color band bounded by the maximum and minimum value of all ensembles over years. Costs beyond the y-axis upper limit indicate that the assessment unit is untappable at that time.

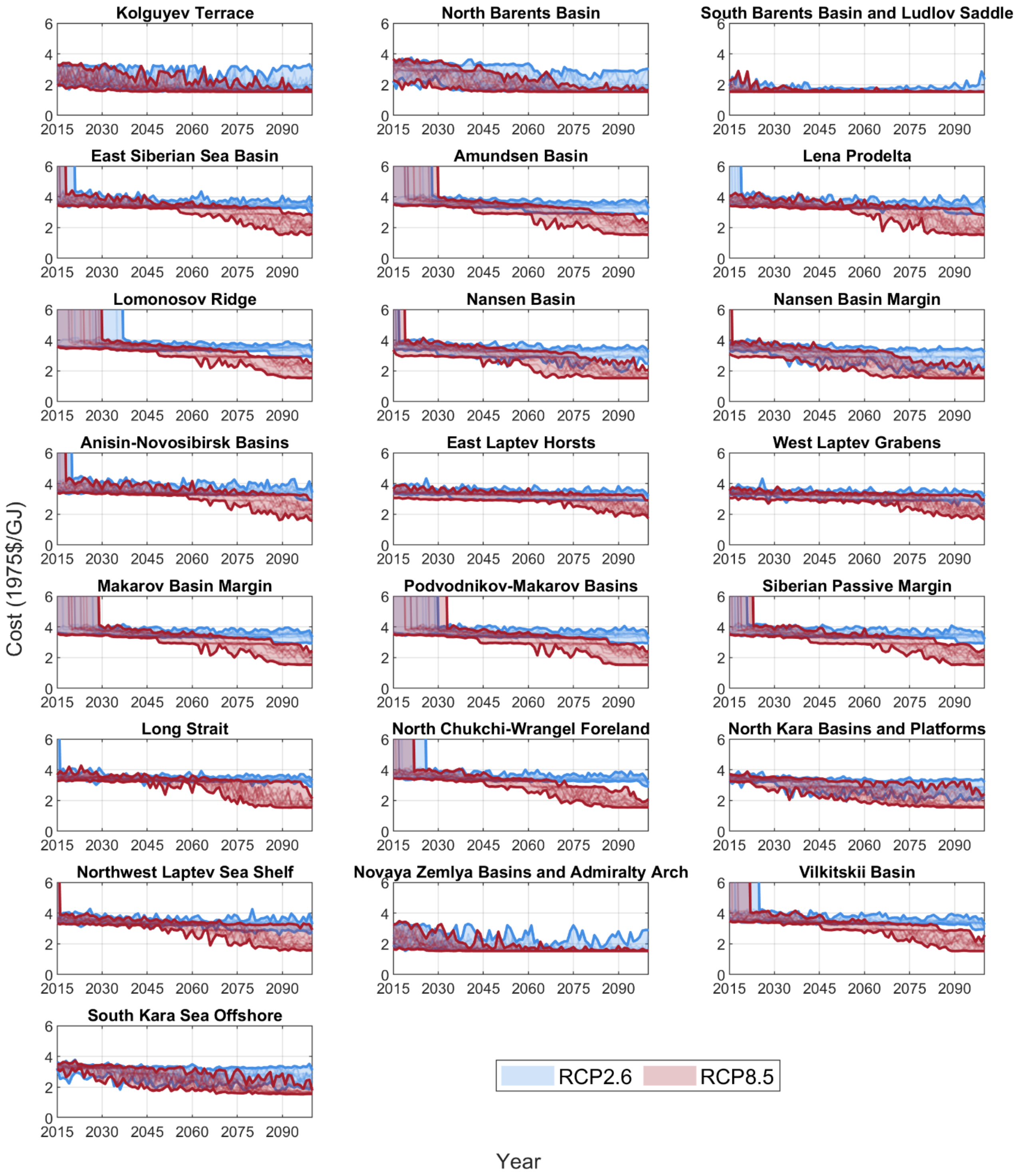

Fig. S17 Extraction costs for Arctic offshore *oil* over 2015-2100 across Assessment Units (AUs) in **Russia**, given sea ice thickness projections from CMIP6 model ensemble under RCP2.6 and 8.5. Each RCP category is shown in a single-color band bounded by the maximum and minimum value of all ensembles over years. Costs beyond the y-axis upper limit indicate that the assessment unit is untappable at that time.

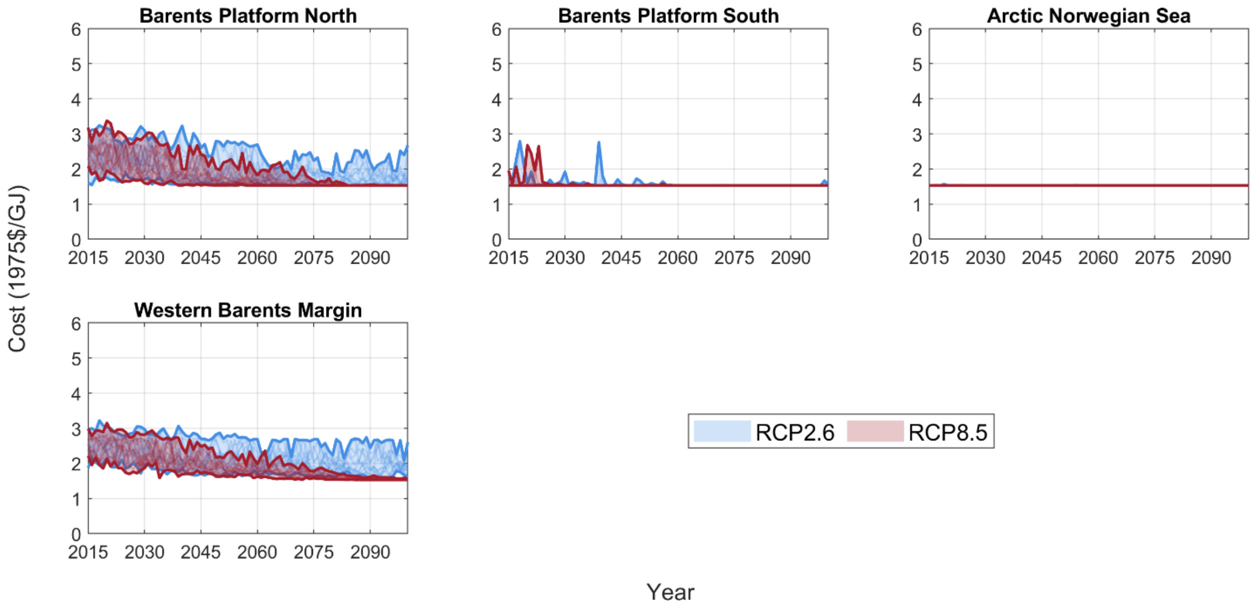

Fig. S18 Extraction costs for Arctic offshore *gas* over 2015-2100 across Assessment Units (AUs) in **Norway**, given sea ice thickness projections from CMIP6 model ensembles under RCP2.6 and 8.5. Each RCP category is shown in a single-color band bounded by the maximum and minimum value of all ensembles over years. Costs beyond the y-axis upper limit indicate that the assessment unit is untappable at that time.

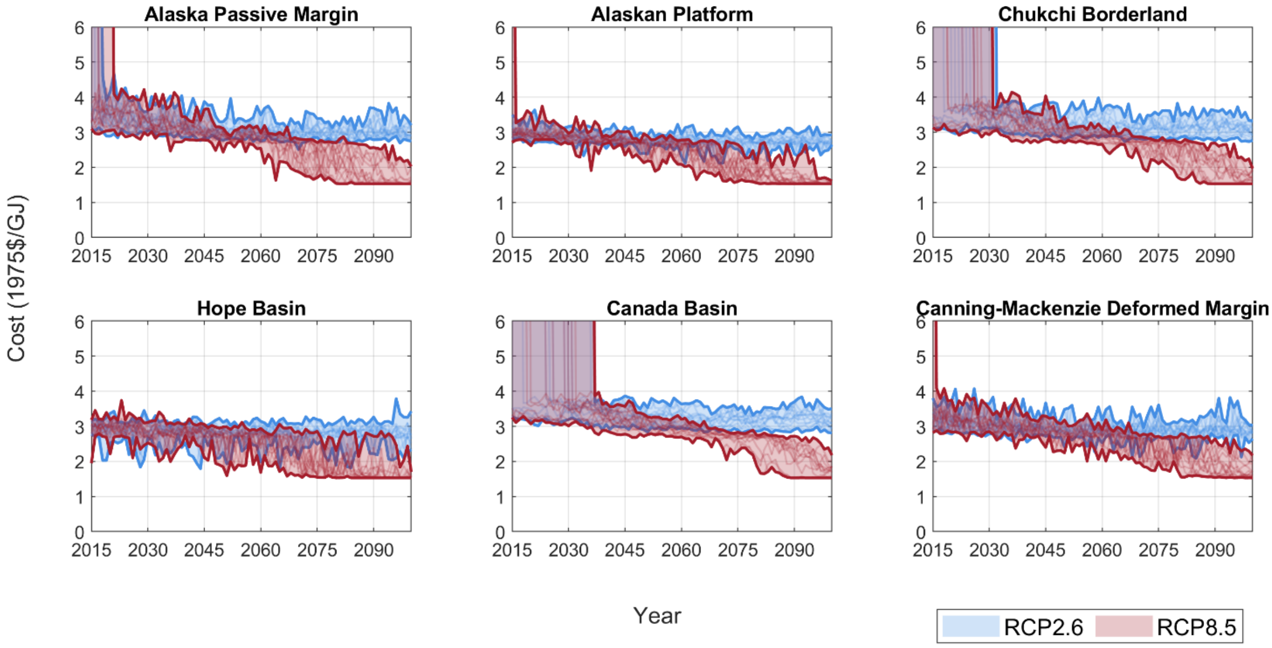

Fig. S19 Extraction costs for Arctic offshore *gas* over 2015-2100 across Assessment Units (AUs) in the **USA**, given sea ice thickness projections from CMIP6 model ensembles under RCP2.6 and 8.5. Each RCP category is shown in a single-color band bounded by the maximum and minimum value of all ensembles over years. Costs beyond the y-axis upper limit indicate that the assessment unit is untappable at that time.

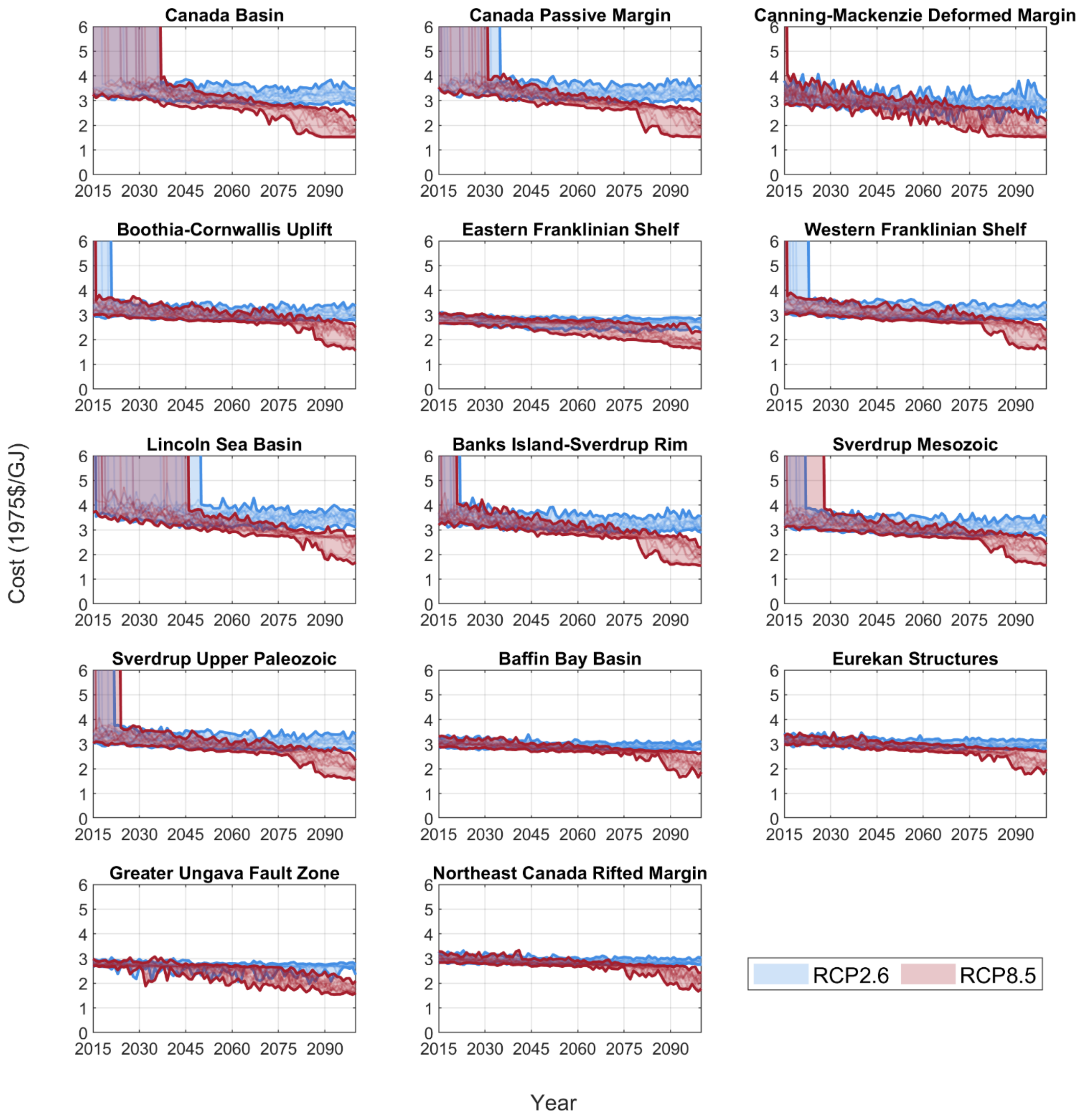

Fig. S20 Extraction costs for Arctic offshore *gas* over 2015-2100 across Assessment Units (AUs) in **Canada**, given sea ice thickness projections from CMIP6 model ensembles under RCP2.6 and 8.5. Each RCP category is shown in a single-color band bounded by the maximum and minimum value of all ensembles over years. Costs beyond the y-axis upper limit indicate that the assessment unit is untappable at that time.

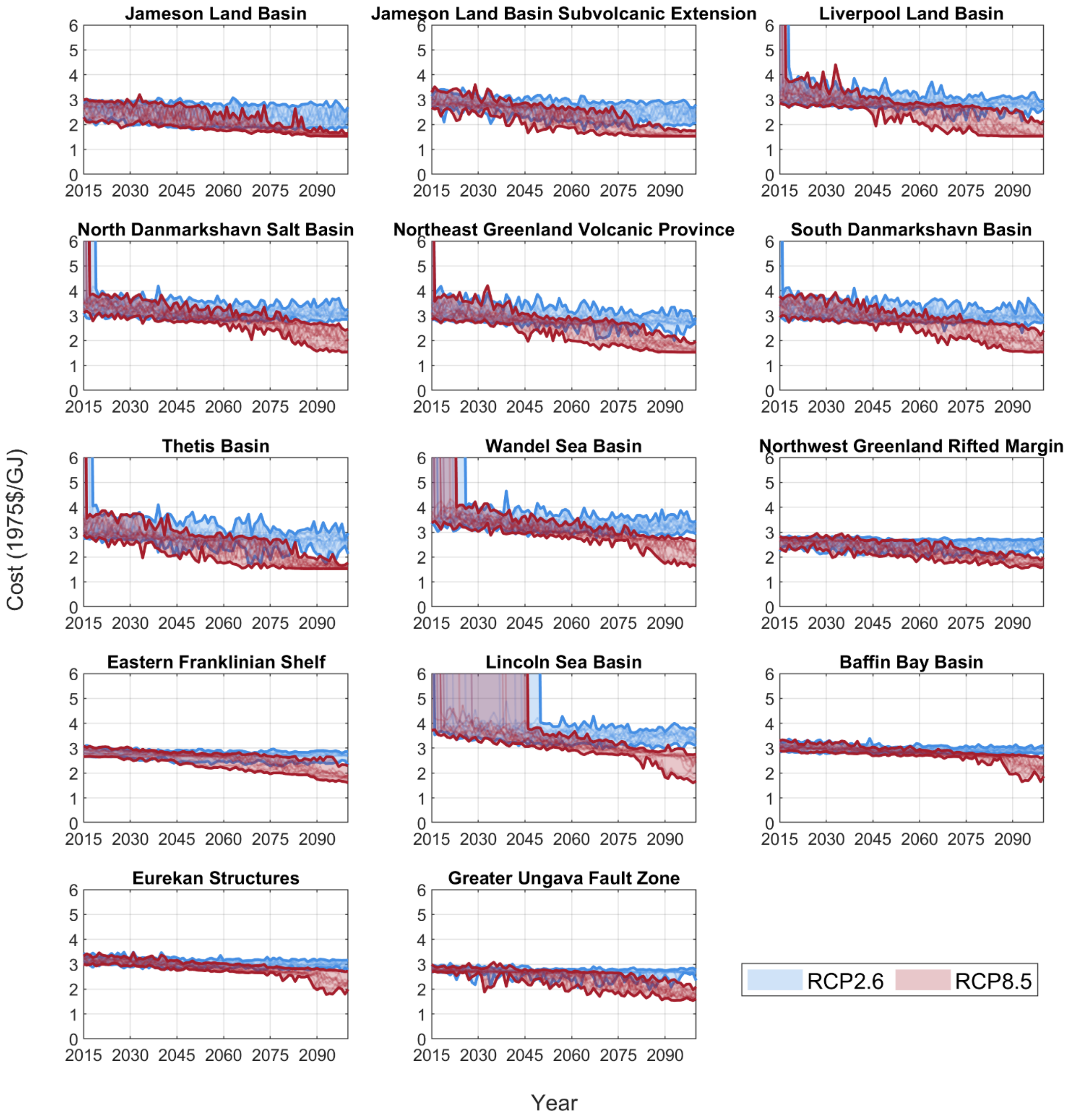

Fig. S21 Extraction costs for Arctic offshore *gas* over 2015-2100 across Assessment Units (AUs) in **Greenland**, given sea ice thickness projections from CMIP6 model ensembles under RCP2.6 and 8.5. Each RCP category is shown in a single-color band bounded by the maximum and minimum value of all ensembles over years. Costs beyond the y-axis upper limit indicate that the assessment unit is untappable at that time.

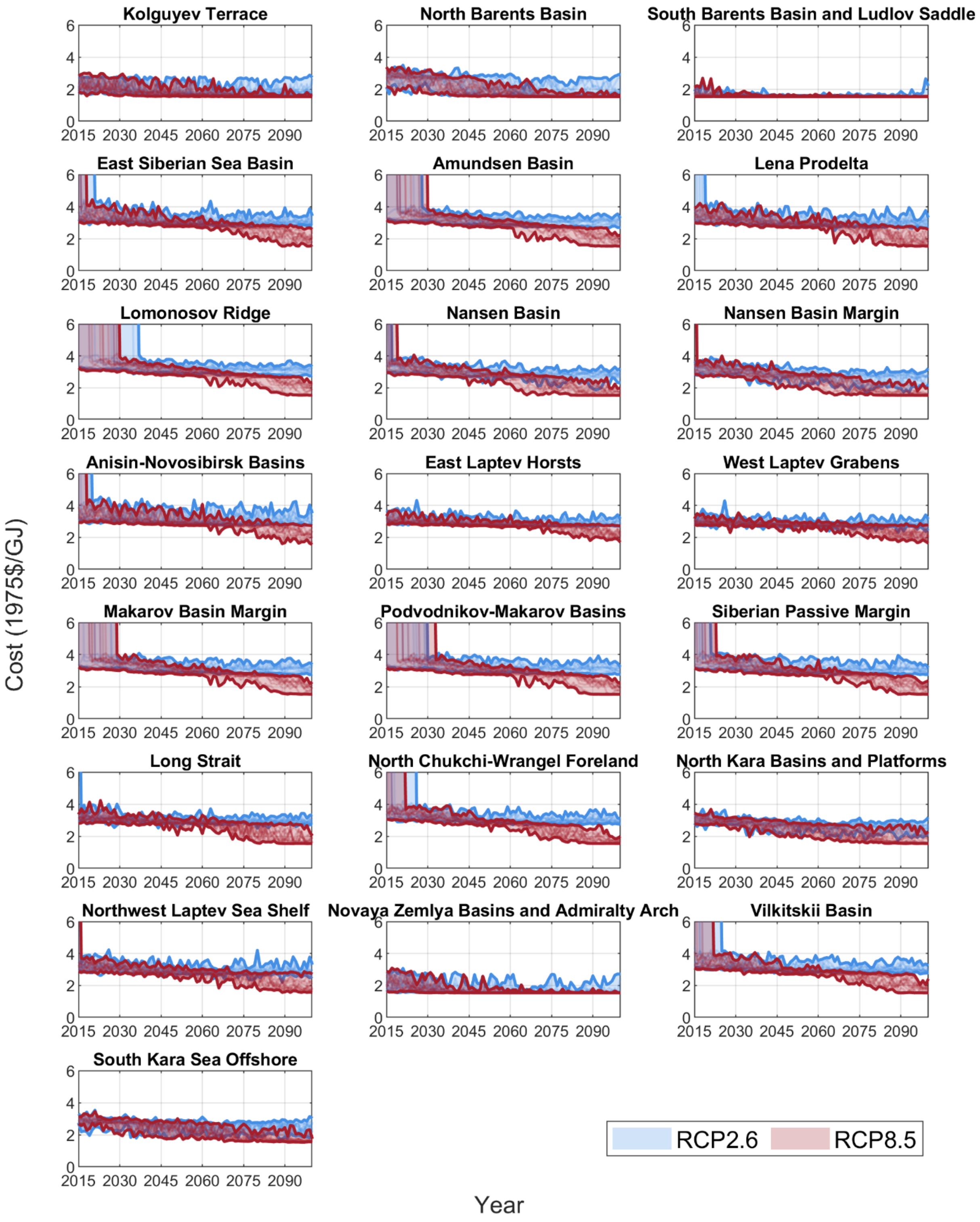

Fig. S22 Extraction costs for Arctic offshore *gas* over 2015-2100 across Assessment Units (AUs) in **Russia**, given sea ice thickness projections from CMIP6 model ensembles under RCP2.6 and 8.5. Each RCP category is shown in a single-color band bounded by the maximum and minimum value of all ensembles over years. Costs beyond the y-axis upper limit indicate that the assessment unit is untappable at that time.

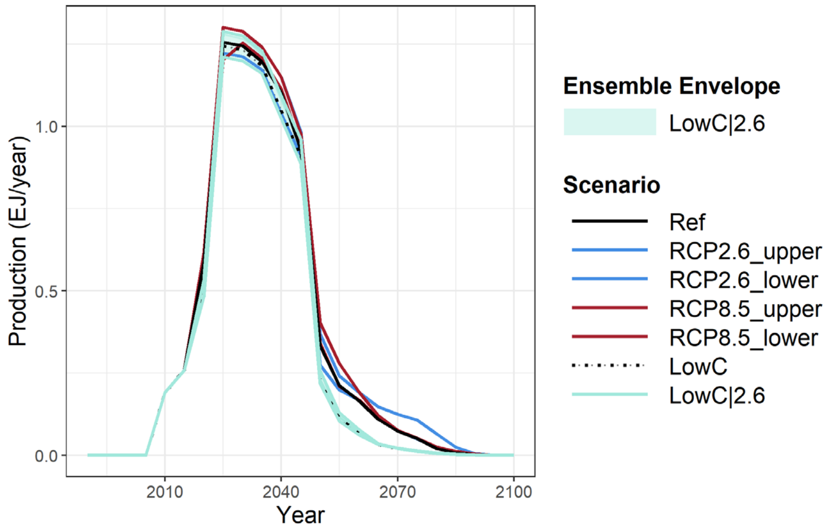

Fig. S23 Arctic offshore gas production in Norway under model scenarios. For scenarios that include 13 ensembles, an upper and lower bound are shown. LowC|2.6 is also shown with a partially transparent ensemble envelope.

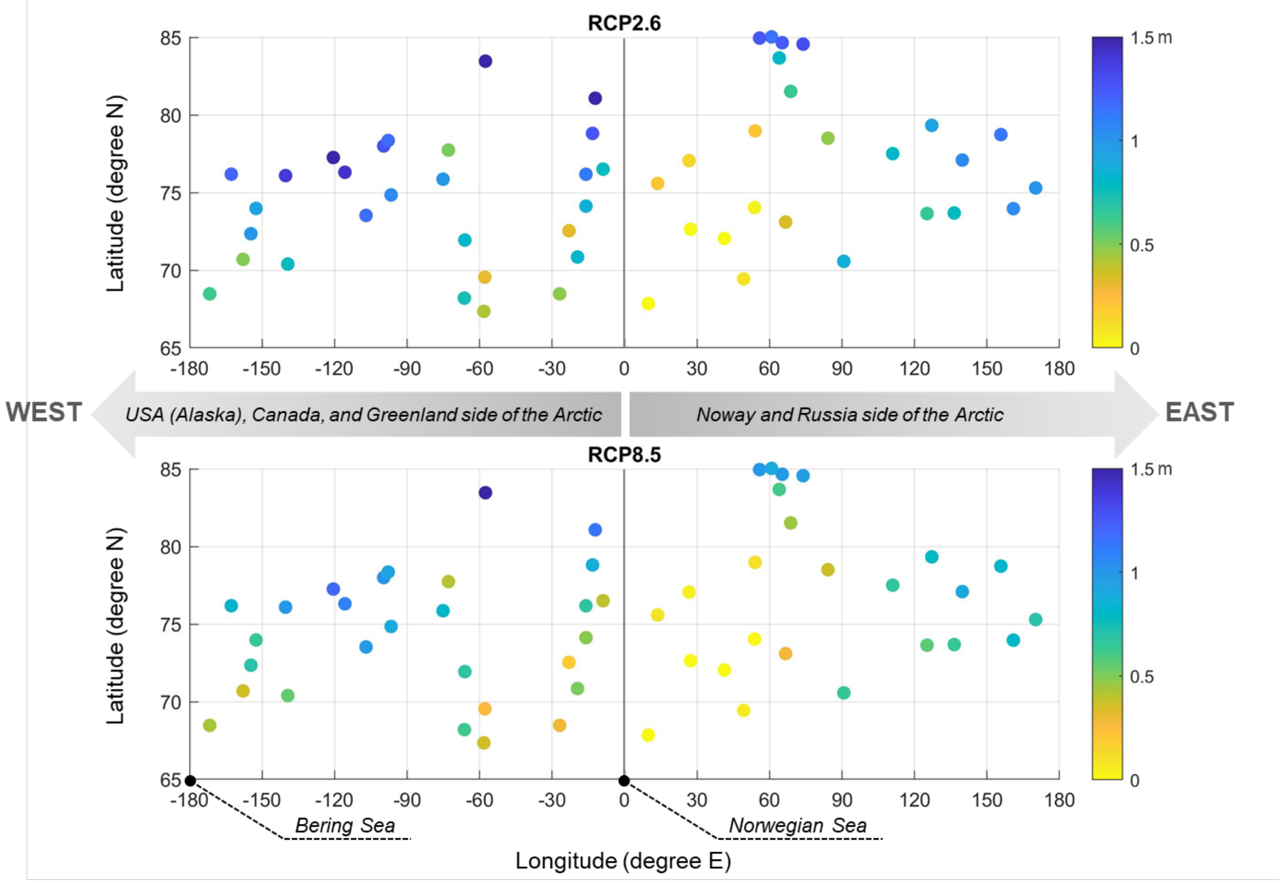

Fig. S24 Sea ice thickness under RCP2.6 and 8.5 in 5-year average around 2050, at offshore assessment units marked by latitude and longitude at the center of each assessment unit. The color represents the sea ice thickness in meters using the average of 13 CMIP6 ensembles explored in this study.

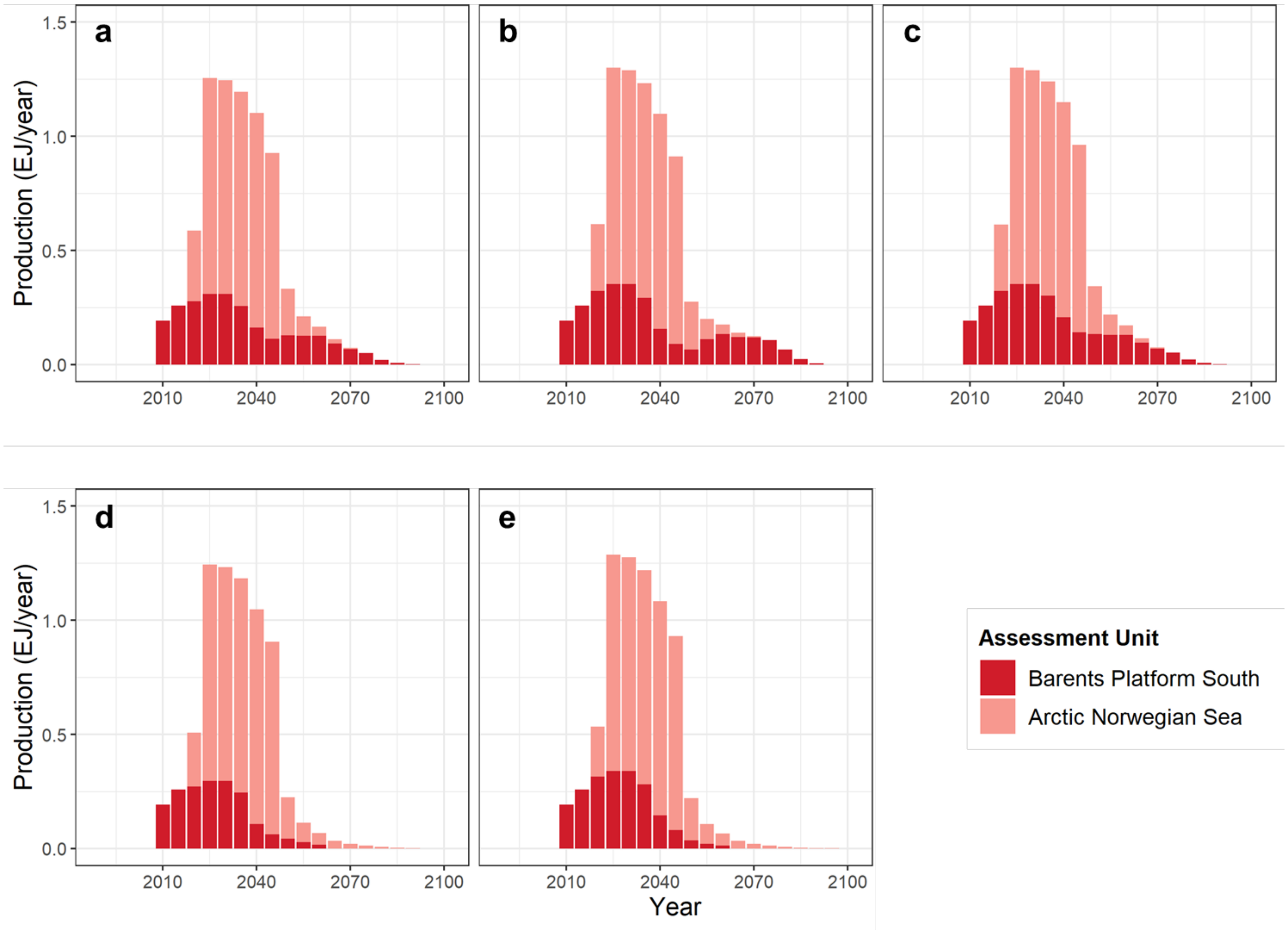

Fig. S25 Arctic offshore gas production aggregated over Assessment Units (AUs) in Norway, under **a**) Ref, **d**) LowC, and for a random ensemble under **b**) RCP2.6, **c**) RCP8.5, and **e**) LowC|2.6.

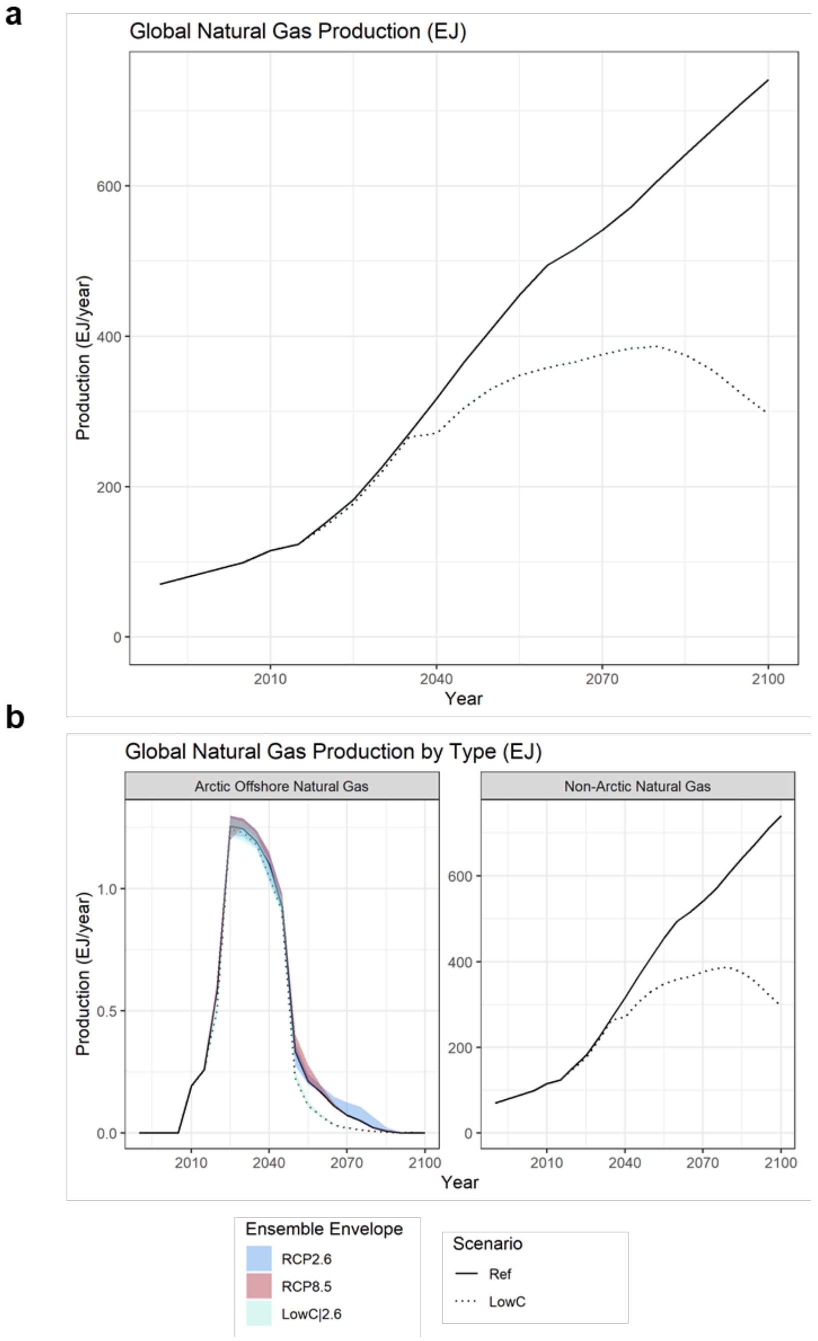

Fig. S26 a) Global gas production as a sum of Arctic offshore natural gas and non-Arctic natural gas over time under model scenarios; **b**) Global gas production by the two types of gas over time under the same model scenarios. Note that the differences between Ref, RCP2.6, and RCP 8.5 are negligible in a), as well as in b) for non-Arctic natural gas.

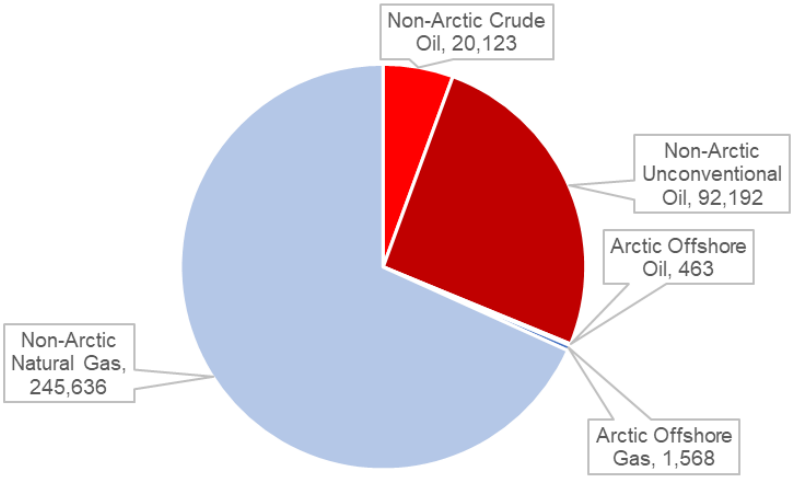

Fig. S27 Discovered and undiscovered (estimated) oil and gas resources in the world. Data from GCAM inputs (publicly available at https://github.com/JGCRI/gcam-core) based on analysis of Rogner (1997). Arctic offshore oil and gas resource potentials are the mean estimates reported by USGS (Bird et al. 2008).

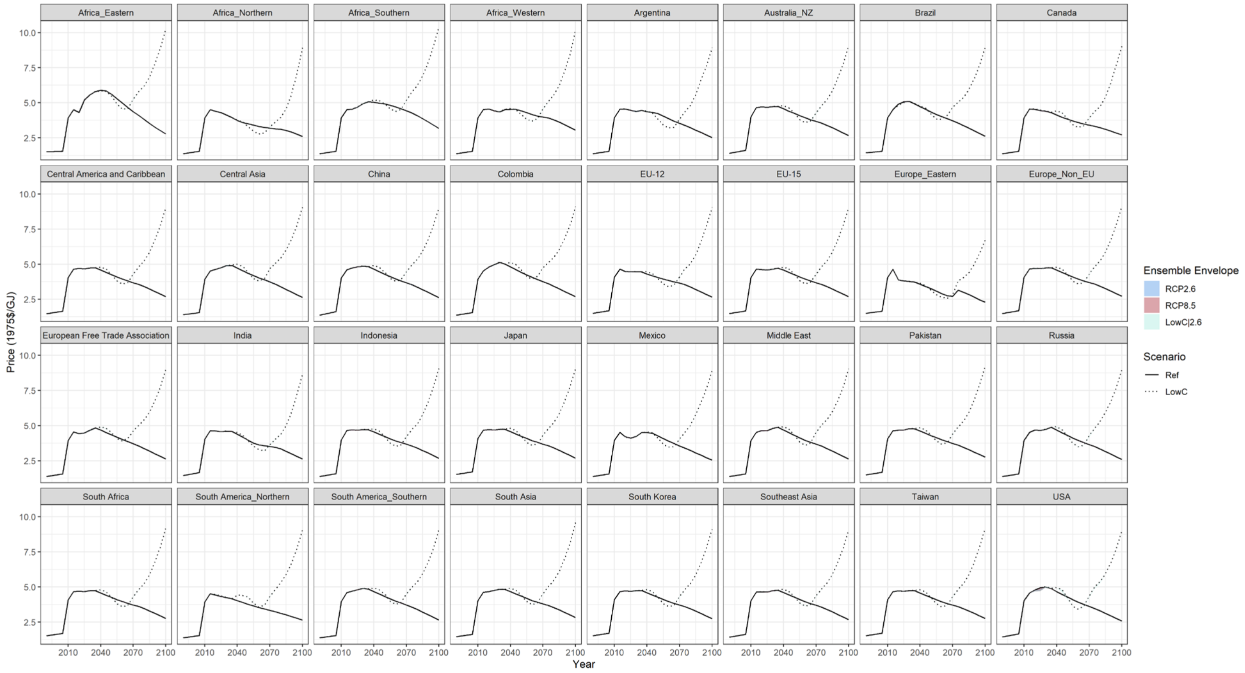

Fig. S28 Regional oil prices under different scenarios. Note that RCP2.6 and RCP8.5 have very similar prices to Ref; LowC|2.6 has very similar prices to LowC; therefore, those prices are overlapped on this figure.

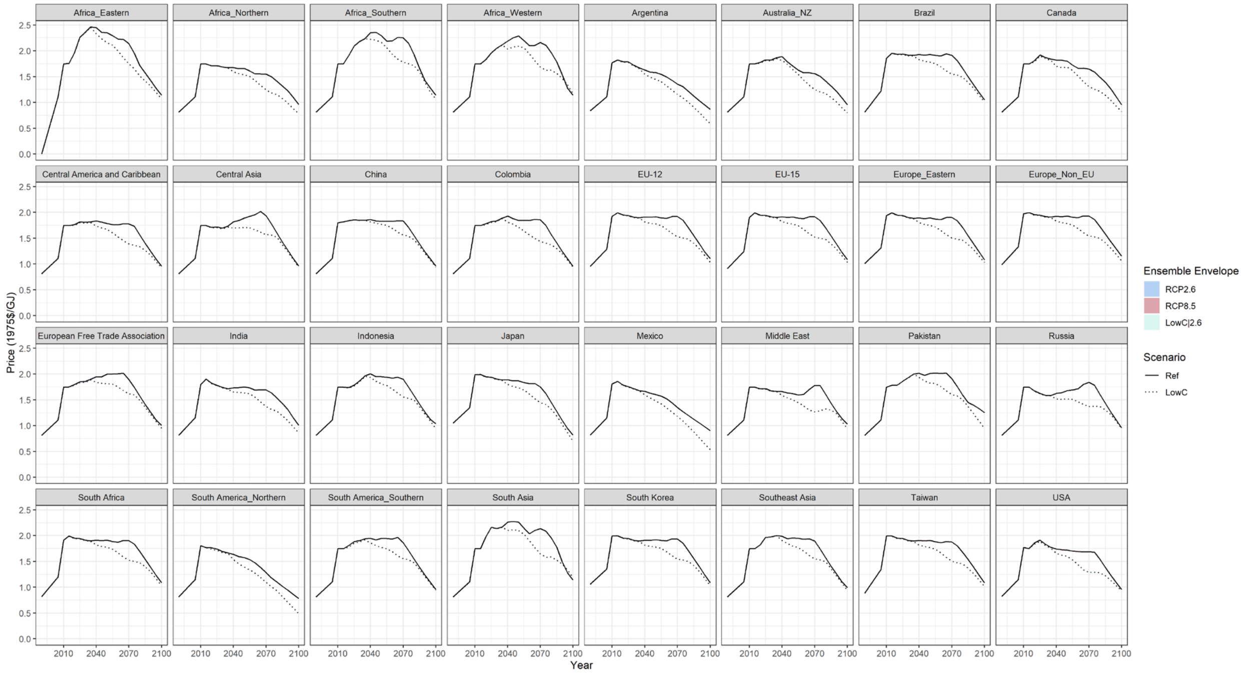

Fig. S29 Regional natural gas prices under different scenarios. Note that RCP2.6 and RCP8.5 have very similar prices to Ref; LowC|2.6 has very similar prices to LowC; therefore, those prices are overlapped on this figure.

Appendix S2

Resource Supply Curves

The AU-level annual extraction costs in the base year (i.e., 2015) are first averaged across all climate ensembles and RCP scenarios, so that a uniform base-year cost is obtained at each AU, from which the future costs start to diverge under different climate ensembles and RCP scenarios. After that, a five-year average of the annual extraction costs centered around the GCAM future periods (i.e., 2020, 2025, …, 2100) are taken and combined with the base-year uniform costs. As a result, given a climate ensemble and an RCP scenario, each AU is associated with an extraction cost, which may vary over time, and a resource potential for oil and gas, respectively.

To generate resource supply curves for GCAM implementation, the AUs are sorted ascendingly by the associated costs within each GCAM region at each GCAM period. The costs and the cumulative resource potential of the sorted AUs are then combined to generate resource supply curves. This implies that within each region the AU with the cheapest resource will be extracted first and so forth. Note that because the AU-level extraction costs are changing over time, the resource supply curves are also changing. It is also possible that, at a different time, a different AU becomes the one with the cheapest resource due to the spatial-temporal variability of sea ice thickness.

References

Bird, Kenneth J., Ronald R. Charpentier, Donald L. Gautier, David W. Houseknecht, Timothy R. Klett, Janet K. Pitman, Thomas E. Moore, Christopher J. Schenk, Marilyn E. Tennyson, and Craig J. Wandrey. 2008. "Circum-Arctic resource appraisal: estimates of undiscovered oil and gas north of the Arctic Circle." In.: U.S. Geological Survey Fact Sheet 2008-3049, 4 p.

Eyring, V., S. Bony, G. A. Meehl, C. A. Senior, B. Stevens, R. J. Stouffer, and K. E. Taylor. 2016. 'Overview of the Coupled Model Intercomparison Project Phase 6 (CMIP6) experimental design and organization', *Geosci. Model Dev.*, 9: 1937-58.

International Energy Agency. 2008. "World energy outlook." In.: IEA/OECD.

Marszałkowski, Mariusz. 2019. 'How much does Russian oil really cost? (ANALYSIS)', Accessed June 21, 2022. https://biznesalert.com/russia-oil-extraction-cost-analysis/.

Petrick, Sebastian, Kathrin Riemann-Campe, Sven Hoog, Christian Growitsch, Hannah Schwind, Rüdiger Gerdes, and Katrin Rehdanz. 2017. 'Climate change, future Arctic Sea ice, and the competitiveness of European Arctic offshore oil and gas production on world markets', *Ambio*, 46: 410-22.

Rogner, H-H. 1997. 'AN ASSESSMENT OF WORLD HYDROCARBON RESOURCES', *Annual Review of Energy and the Environment*, 22: 217-62.
